# Sustainable Remediation of Landfill Leachate Through Sludge-Based Adsorbents: A Critical Review of Synthesis, Performance, and Circularity

**DOI:** 10.3390/molecules31162905

**Published:** 2026-08-20

**Authors:** Maria Râpă, Adrian Bîldea, Ecaterina Matei, Alina-Cornelia Ion, Cristian Predescu

**Affiliations:** 1 Faculty of Materials Science and Engineering, National University of Science and Technology Politehnica Bucharest, 060042 Bucharest, Romania; maria.rapa@upb.ro (M.R.); ecaterina.matei@upb.ro (E.M.); cristian.predescu@upb.ro (C.P.); 2 Biotechnical Systems Engineering Doctoral School, National University of Science and Technology Politehnica Bucharest, 313 Splaiul Independentei, 060042 Bucharest, Romania

**Keywords:** sewage sludge, landfill leachate, adsorption mechanisms, resource recovery, biochar, circular economy

## Abstract

The complex composition of landfill leachate, characterized by high concentrations of refractory organic matter, ammonium nitrogen, and heavy metals, requires efficient and sustainable treatment technologies. Recently, sludge-based adsorbents (SBAs) obtained from wastewater treatment plants (WWTPs) sludge have emerged as promising alternatives to the conventional activated carbon materials for landfill leachate. This review critically evaluates the recent literature related to the transformation of sludge waste and municipal solid waste-based adsorbents into high-efficiency SBAs as a circular economy strategy for leachate remediation. The correlation of the physicochemical characteristics of landfill leachate with the properties of SBAs to enhance the removal of specific contaminants is discussed. The treatment strategies for landfill leachate including physicochemical, biological, and integration of those are summarized. By correlating adsorption performance with SBAs’s properties and circular economy principles, this review identifies the key knowledge gaps and provides guidance for the development and large-scale implementation of sustainable leachate treatment technologies. Furthermore, the prospects for the application of SBAs in the closed-loop landfill leachate treatment system are addressed.

## 1. Introduction

With the global population reaching approximately 8.16 billion in 2024 and projected to rise to 10 billion in the twenty-second century, the demand for food, energy, and material resources is expected to increase substantially [1]. Consequently, growing consumption patterns are generating unprecedented quantities of waste, posing significant challenges for sustainable resource management and environmental protection. To address these growing waste streams, several waste management strategies have been proposed, including landfilling, recycling, composting, incineration, and integrated systems that combine incineration with full landfill gas (LFG) recovery. Among these options, landfilling remains the most widely adopted method in both developed and developing countries because of its relatively low cost and modest technical requirements [2]. However, one of the major drawbacks of landfilling is the generation of leachate, a toxic and highly concentrated liquid effluent produced by the percolation of rainwater through disposed solid waste, the inherent moisture content of the waste, and the decomposition of organic materials within the landfill [3,4]. Landfill leachate contains a complex mixture of contaminants, including total dissolved solids (TDS), dissolved organic matter measured as chemical oxygen demand (COD), hazardous organic pollutants (polycyclic aromatic hydrocarbons (PAHs), antibiotics, pesticides, and endocrine-disrupting chemicals), inorganic compounds, volatile fatty acids (VFAs), macro-components such as ammonium nitrogen (NH_4_^+^–N), and microplastics, all of which pose significant risks to ecosystems and human health [2,3,4,5,6,7,8,9,10,11,12,13,14]. In addition, landfill leachate may contain high concentrations of heavy metals such as Cd, Cr, Cu, Pb, Ni, Zn, and Hg ions originating from the deposition of airborne suspended particulate matter onto the landfill surface, with subsequent wash-off into the leachate [15]. Landfill leachate may exhibit a dark color due to the humic and fulvic compounds and elevated concentrations of Fe. Besides the need for technologies to remove recalcitrant pollutants from landfill leachate and achieve water reclamation, landfill leachate also represents the potential for recovery of valuable resources such as humic substances, trace metals, nutrients (N and P), and energy [16]. Recently, a study employing machine-learning-enhanced landfill gas modeling has demonstrated that the hybrid waste-to-energy approaches can simultaneously reduce CH_4_ emissions, increase renewable energy production, and improve the economic sustainability of municipal solid waste (MSW) management [17]. Recovering CH_4_ from landfill gas offers substantial potential for renewable energy generation, while simultaneously mitigating GHG emissions [18,19,20]. For example, Bensmain et al. [18] reported that approximately 178 m^3^ of biogas could be generated from one ton of waste collected from three operational landfills in Algeria, corresponding to an energy potential of approximately 1070 kWh.

However, landfills raise additional environmental concerns, particularly regarding greenhouse gas (GHG) emissions. Methane (CH_4_) is recognized as a significant greenhouse gas, exhibiting a global warming potential 21–28 times higher than that of CO_2_ [19]. The importance of CH_4_ mitigation is underscored by the latest findings of the Intergovernmental Panel on Climate Change (IPCC). According to the Sixth Assessment Report (AR6), the effective radiative forcing of CH_4_ increased from 0.48 ± 0.10 W/m^2^ for the period 1750–2011 to 0.54 ± 0.11 W/m^2^ for 1750–2019, highlighting the contribution of CH_4_ to increasing climate change [21]. The GHG emissions resulting from a case study of MSW landfills with a high food waste content (HFWC) showed that the landfill generated a total of 21.23 million tons of CO_2_-eq emissions between 2007 and 2022, with an average of 1.03 tons of CO_2_-eq per ton of MSW [22].

Landfill leachate is commonly co-treated with municipal wastewater in wastewater treatment plants (WWTPs) [9,23]. WWTPs generate considerable quantities of waste-activated sludge, a major by-product of biological wastewater treatment. The issue of utilizing sludge from wastewater treatment has become a major concern due to the expansion of wastewater treatment infrastructure and the rising volumes of sludge produced. The sludge resulting from these processes contains significant amounts of organic matter and nutrients that can be utilized in agriculture. However, it may also contain pathogens, heavy metals, and other contaminants that pose risks to human health and the environment if not managed properly [24,25,26,27]. Adsorption, anaerobic digestion, and thermal treatment are widely applied technologies to stabilize and valorize sewage sludge [25,28,29,30,31,32]. More recently, the carbonization of sewage sludge has emerged as a sustainable management strategy, converting it into biochar, a low-cost adsorbent suitable for landfill leachate treatment [29,33,34]. This approach simultaneously addresses the management of both sewage sludge and landfill leachate while promoting circular economy principles by transforming one waste stream into a valuable resource for the treatment of another.

Activated carbon (AC) and zeolite are the most widely used commercial adsorbents for the removal of organic compounds and color from landfill leachate, showing excellent adsorption performance, broad-spectrum contaminant removal capability, and the absence of secondary treatment by-products [35,36,37]. AC can be prepared from sewage sludges through high-temperature carbonization followed by physical or chemical activation, or by direct chemical activation [38]. However, AC and zeolite applications are limited due to the high production cost, energy-intensive regeneration process, and substantial carbon footprint. Several studies have also investigated the partial replacement of AC with natural adsorbents to reduce the treatment costs while maintaining satisfactory removal performance [9,23,39,40,41,42]. Nevertheless, their practical applications remain limited by lower adsorption capacity, variability in physicochemical properties, potential contaminant leaching, and reduced adsorption efficiency after repeated regeneration cycles. Also, the GHG emissions associated with AC production vary considerably depending on the feedstock and activation method used [43].

Table 1 provides a comparison of the previous reviews focused on individual sludge-derived materials, such as biochar or AC, or on broader circular economy strategies for food waste and wastewater valorization.

The reviewed papers highlight several gaps that hinder the commercial-scale implementation of sludge-based activated carbon, including the lack of standardized processing techniques, insufficient regeneration studies, and the need for comprehensive life-cycle assessment. However, none has comprehensively compared the characteristics of landfill leachate, sludge-based adsorbents (SBAs) derived from WWTPs, and appropriate technologies for the effective removal of the complex pollutants present in landfill leachate [44,45,46].

This review summarizes the most recent studies on the valorization of sewage sludge into biochar, activated carbon, and ash-derived sorbents for the treatment of landfill leachate, to remove organic matter, ammonium, heavy metals, antibiotics, and other emerging contaminants. The integration of sewage sludge into landfill leachate management represents a waste-to-resource strategy that addresses two interconnected environmental challenges. By applying this strategy, the burdens associated with the disposal of sewage sludge are reduced, while providing a sustainable and cost-effective solution for the remediation of leachate from landfill, thereby promoting resource recovery and advancing the principles of the circular economy.

## 2. Methodology of Research

To evaluate research trends in SBAs for the treatment of landfill leachate, a literature search was conducted in the ScienceDirect and Web of Science databases covering publications from 2016 to 2026. The search included both original research and review articles using the following keyword combinations: “adsorbents for leachate treatment,” “sludge adsorbents for leachate treatment,” and “nanoparticle-based sludge adsorbents for leachate”. A total of 1030 publications were identified through the database search. However, the term “sludge” broadly refers to solids generated from different industrial, municipal solid waste, and wastewater treatment processes. After removing 235 duplicate records, 795 publications remained for title, abstract, and relevant data screening. Subsequently, by excluding articles that did not investigate landfill leachate and adsorbents that were not derived from sludge, 680 articles were excluded. Following the application of the eligibility criteria, only 115 studies that investigated the preparation, characterization, or application of sludge-derived adsorbents for the treatment of landfill leachate were kept for full-text assessment. From each study, information about the characteristics of landfill leachate, preparation of the adsorbent, characterization techniques, adsorption conditions, removal efficiencies, regeneration performance, and adsorption mechanisms was extracted.

**Figure 1:**
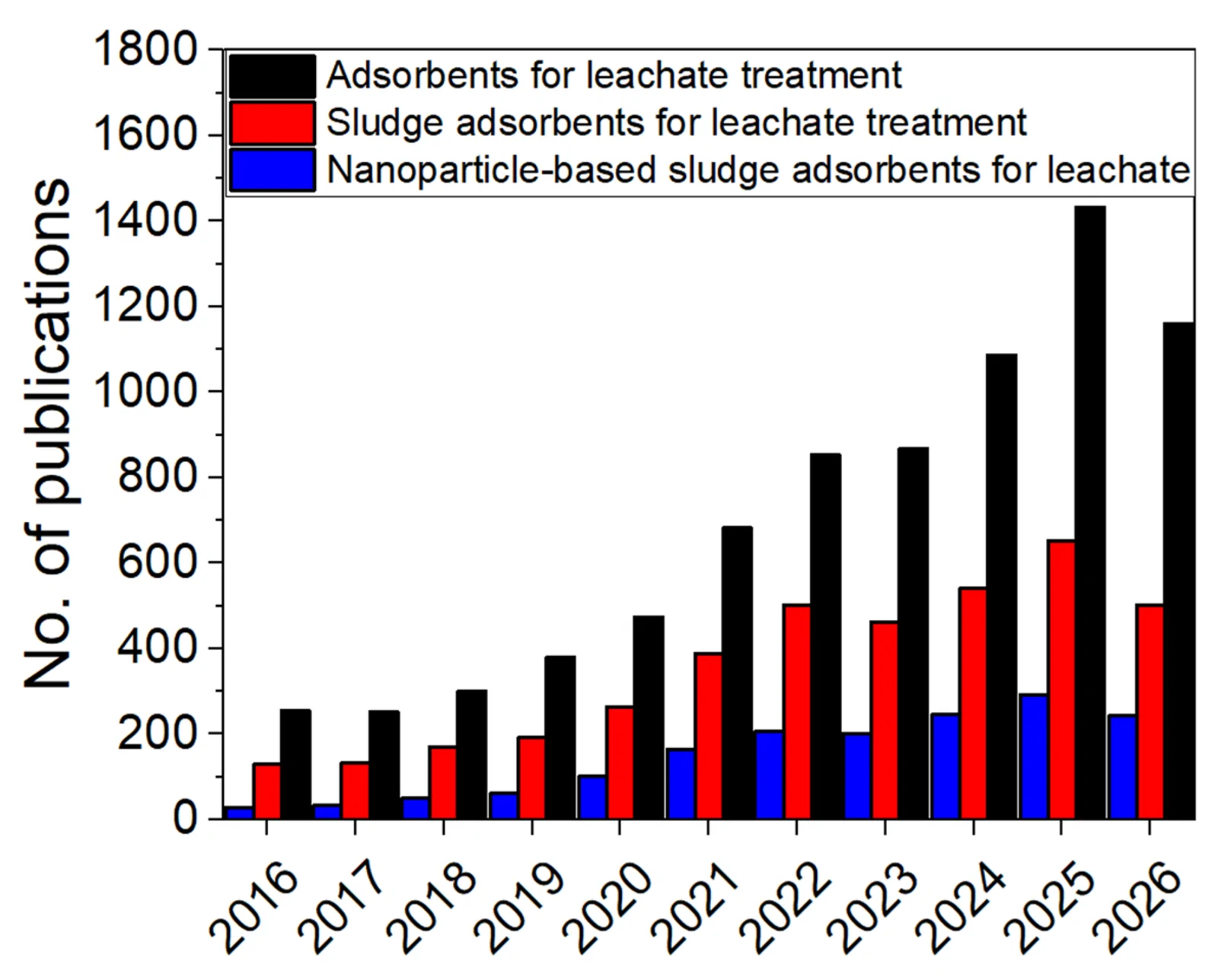
Illustration of the publications that appeared on the ScienceDirect database, from 2016 to 2026.

Figure 1 shows the number of publications recorded for each keyword, which illustrates the evolution of research activity in this field over the selected period.

**Figure 2:**
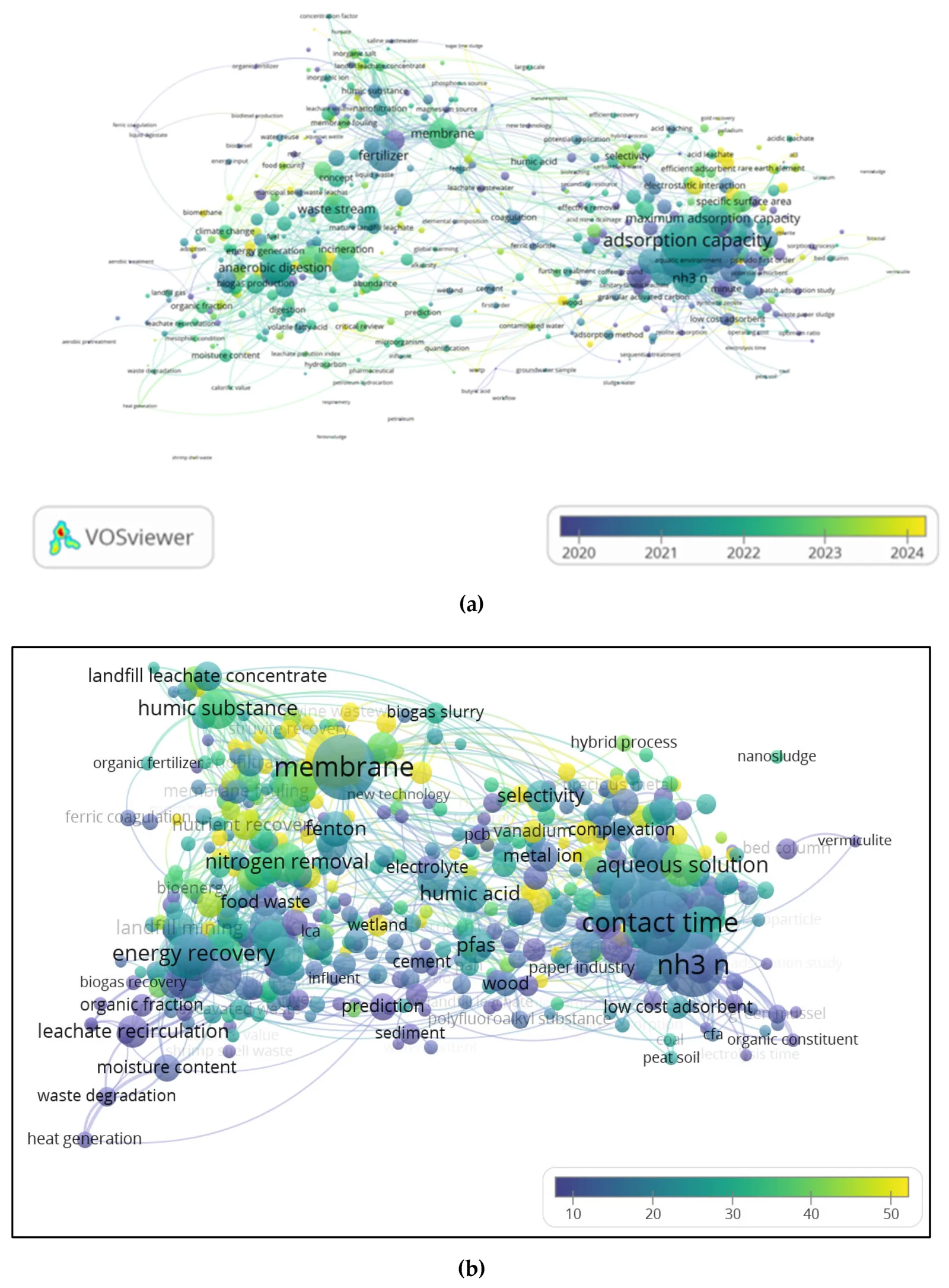
Average of publications per year (**a**) and average of citations per year (**b**) according to Web of Science Core Collection, from 2016 up to now, using keywords: “sludge adsorbents for leachate treatment”; “adsorbents used for leachate treatment”; and “resource recovery from landfill leachate”.

Bibliometric analysis was also performed using VOSviewer (version 1.6.20). A minimum keyword occurrence threshold of five was applied, resulting in the identification of twelve clusters visualized based on the average publications per year (Figure 2a) and the average citations per year (Figure 2b). The keyword co-occurrence network reveals that the research on landfill leachate is primarily focused on membrane technologies, adsorption, nitrogen removal, energy recovery, and contaminant remediation, highlighting the multidisciplinary nature of the field. Overlay visualizations indicate a growing research emphasis on sustainable treatment strategies, including low-cost adsorbents, hybrid processes, and energy recovery, while advanced treatment technologies continue to receive the greatest scientific impact. Despite these advances, sewage SBAs remain underrepresented in the bibliometric network, revealing a significant knowledge gap and emphasizing the need for a comprehensive review of their development, adsorption mechanisms, and application in sustainable landfill leachate treatment.

## 3. Characteristics of Landfill Leachate

### 3.1. Composition of Landfill Leachate

**Figure 3:**
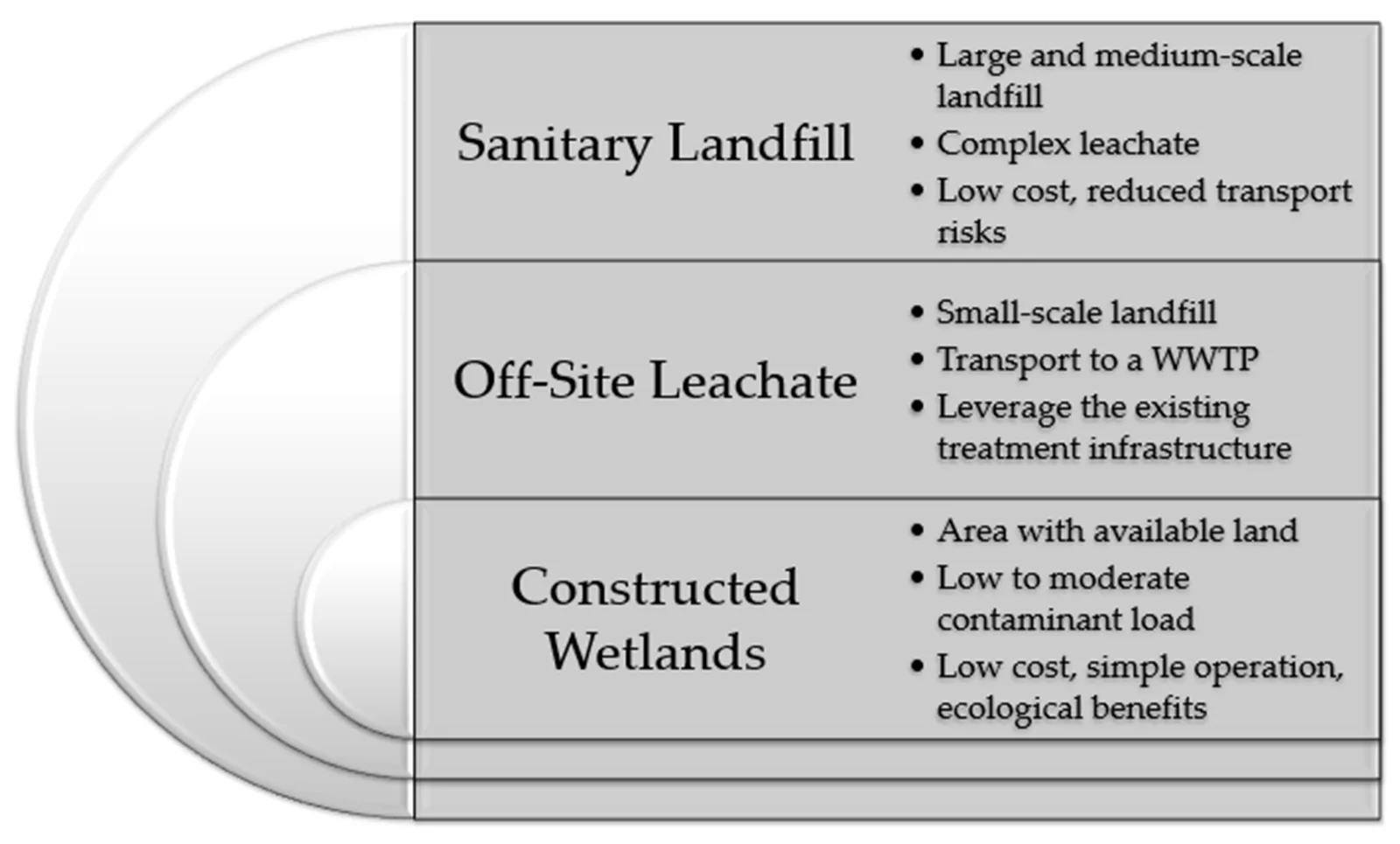
The treatment options for landfill leachate.

The treatment of landfill leachate can be carried out using systems located on the landfill site itself, off-site treatment facilities, or in constructed wetlands (CWs), depending on the characteristics of the leachate, regulatory requirements, and economic considerations (Figure 3) [9,54,55,56,57].

Sanitary landfills treat complex landfill leachate and offer advantages over other approaches due to the low cost and reduced risks associated with transport. For small-scale landfills, off-site treatment is the best solution, which usually involves treating the leachate near a municipal WWTP. CWs represent an attractive solution in areas where land is available and are used for polishing treated effluent or treating leachate with a relatively low contaminant load.

The composition of landfill leachate varies considerably among landfill sites, and it depends on the waste characteristics, geographical location, landfill age, and environmental factors [58]. Consequently, substantial variations in the leachate quality have been reported worldwide. For example, high COD values of 16,000 and 90,000 mg/L were reported in sanitary landfill leachate from Turkey [59] and a pilot landfill leachate from China [60], a concentration of Fe of 8.659 mg/L and color of 4539 Pt-Co were found in Malaysian leachate [39], and NH_4_^+^ ≈ 4827 mg/L was reported in Moroccan leachate [61].

Given this variability, reliable predictive tools are essential for the efficient monitoring and management of leachate. In this regard, Azadi et al. [62] developed two expert systems based on Artificial Neural Network (ANN) and Principal Component Analysis-M5P (PCA-M5P) models to predict the COD load of laboratory-scale landfill leachate. Both models achieved excellent predictive performance (R > 0.98), with the ANN model outperforming the PCA-M5P model (MAPE = 4% versus 12%), demonstrating the potential of machine learning for the cost-effective monitoring and management of leachate.

Based on the age of the landfill, leachate is commonly classified as young leachate (originating from landfills operating for less than five years), intermediate leachate (5–10 years), and stabilized (old) leachate (more than ten years) [63,64]. A high biodegradability is given by the young leachate, for which the BOD_5_/COD ratio is greater than 0.5. Stabilized leachate is characterized by a BOD_5_/COD ratio <0.1, a pH value around 8, and a high accumulation of recalcitrant pollutants, meaning a low biodegradability [7,63].

The physicochemical characterization of landfill leachate typically includes measuring pH, electrical conductivity (EC), COD, BOD_5_, total Kjeldahl nitrogen (TKN), NH_4_^+^-N, total phosphorus (TP), total suspended solids (TSS), total dissolved solids (TDS), and color (Pt-Co). The adsorption performance of SBAs is commonly evaluated using adsorption isotherms (Langmuir and Freundlich) and kinetic models, which effectively predict the adsorption mechanisms and rates. The heavy metal concentrations in the leachate are usually determined by Inductively Coupled Plasma Optical Emission Spectroscopy (ICP-OES) [4,6,65].

### 3.2. Regulatory Frameworks for Landfill Leachate and Sludge

The increasing generation of landfill leachate is associated with substantial environmental and economic challenges. In 2020, the global direct cost of waste management was estimated at approximately US$252 billion [66]. Without urgent and effective improvements in waste management practices, this cost is projected to increase substantially, reaching approximately US$640.3 billion annually by 2050 [66]. In response to these growing challenges, regulatory frameworks for waste, landfill leachate, wastewater, and sludge management have been developed in many countries to protect human health and the environment. However, the regulatory requirements and discharge limits vary considerably among countries.

In the European Union (EU), Directive (EU) 2018/850, which amended Directive 1999/31/EC on the landfill of waste, reinforces requirements for the prevention, collection, monitoring, and treatment of landfill leachate to protect surface water and groundwater [67]. Directive 2000/60/EC establishes a comprehensive framework for the protection of inland surface waters, transitional waters, coastal waters, and groundwater [68]. In addition, the Sewage Sludge Directive 86/278/EEC regulates the use of sewage sludge in agriculture to prevent harmful effects on soil, vegetation, animals, and human health while promoting its safe use as a fertilizer [69]. Regulation (EU) 2020/741 establishes minimum requirements for the quality and monitoring of reclaimed water, together with provisions for risk management, to ensure its safe use within an integrated water management framework [70]. For agricultural irrigation, the regulation specifies quality requirements for different reclaimed-water classes; for example, for Class A reclaimed water, these include BOD_5_ ≤ 10 mg/L, TSS ≤ 10 mg/L, and turbidity ≤ 5 NTU [70]. Collectively, these legislative instruments support waste reduction, resource recovery, water protection, and the limitation of adverse impacts on soil and ecosystems. However, despite the comprehensive EU framework for landfill management, water protection, and sewage sludge use, no harmonized EU-wide numerical discharge limits have been established specifically for contaminated landfill leachate.

In the United States, 40 CFR Part 445, “Landfills Point Source Category”, is the primary federal regulation under the U.S. EPA Effluent Guidelines that establishes wastewater discharge standards for landfill facilities. Subpart B, concerning RCRA Subtitle D non-hazardous waste landfills, specifies effluent limitations achievable through the application of the best practicable control technology currently available (BPT) [71]. The Resource Conservation and Recovery Act (RCRA) provides a regulatory framework for the management of hazardous waste from “cradle to grave” [72], while the Clean Water Act regulates point-source wastewater discharges to surface waters through the National Pollutant Discharge Elimination System (NPDES) [73]. However, the NPDES does not establish a single, universally applicable set of numerical discharge limits. Instead, effluent limits for specific parameters are determined on a facility-specific basis, considering factors such as the type of industry, wastewater discharge characteristics, and the condition of the receiving water body [73].

In Canada, there is no uniform, legally binding national set of numerical effluent limits specifically applicable to landfill leachate. Instead, a multi-level regulatory framework is implemented, whereby federal, provincial, and municipal authorities establish requirements through applicable regulations, approvals, and permits. For example, requirements concerning CH_4_ emissions from certain landfills are established under SOR/2025-279 [74]. In Ontario, the “Landfill Standards Guideline” establishes regulatory and approval requirements for the design, operation, closure, and post-closure care of new or expanding non-hazardous waste landfilling sites [75]. This guideline is intended to assist landfill owners, consultants, the public, and other interested stakeholders in understanding the applicable regulatory and approval requirements [75].

In Asia, regulatory approaches on controlling landfill-related pollutants and managing treatment residuals also vary among countries. In China, recent regulatory developments, including CJJ/T 150-2023 [76] and GB 16889-2024 [77], place increasing emphasis on concentrate control and integrated landfill-leachate management [78,79]. In India, the environmentally sound management of hazardous and other wastes is primarily governed by the “Hazardous and Other Wastes (Management and Transboundary Movement) Rules, 2016”.

In South Africa, the environmental regulation is supported by a comprehensive legislative framework that includes the National Environmental Management Act (NEMA), National Environmental Management: Waste Act (NEMWA), National Water Act, and Air Quality Act. Nevertheless, significant enforcement challenges remain, including inadequate enforcement tools, limited institutional capacity, and, in some cases, insufficient political commitment. These factors can hinder effective environmental governance and the implementation of regulatory requirements [80].

Beyond landfill-leachate regulation, the management and beneficial use of WWTP sludge and sludge-derived materials are also governed by various international and national regulatory frameworks. Currently, neither the EU nor the U.S. EPA has established specific regulations governing the use of sewage-sludge-derived biochar in wastewater treatment [81]. Instead, such materials are generally addressed under existing waste-management or biosolids frameworks. China has begun developing relevant standards, including GB/T 33891–2017 [82], which addresses biochar applications in soil remediation [81].

Table 2 summarizes discharge limits for the treated landfill leachate reported for the USA, North Africa, and Asia.

Table 2 demonstrates that no uniform global approach exists for landfill-leachate discharge regulation. The USA emphasizes both daily and monthly discharge performance, while China and Malaysia provide relatively extensive requirements for heavy metals. Morocco, in contrast, places greater emphasis on conventional organic and nutrient parameters in the selected regulatory framework. Compared with the other countries, the Chinese standard therefore provides relatively comprehensive coverage of both organic pollutants and heavy metals. Notably, the Cr(VI) limit of 0.05 mg/L is considerably lower than the U.S. maximum daily value of 1.1 mg/L and monthly average value of 0.46 mg/L, indicating a substantially stricter requirement for this parameter.

As discharge standards become increasingly stringent worldwide, landfill-leachate treatment is evolving beyond conventional bulk pollutant removal toward integrated approaches that emphasize risk management, low-carbon operation, and effective management of treatment residuals. Recent regulatory developments further emphasize concentrate control and integrated leachate management [78,79]. Nevertheless, substantial differences remain among national and regional regulatory frameworks, particularly regarding numerical discharge limits, treatment requirements, and the management of concentrated residual streams. This regulatory heterogeneity highlights the need for continued development and harmonization of standards for the management and treatment of landfill leachate.

### 3.3. Methods Used for the Management of Landfill Leachate

**Figure 4:**
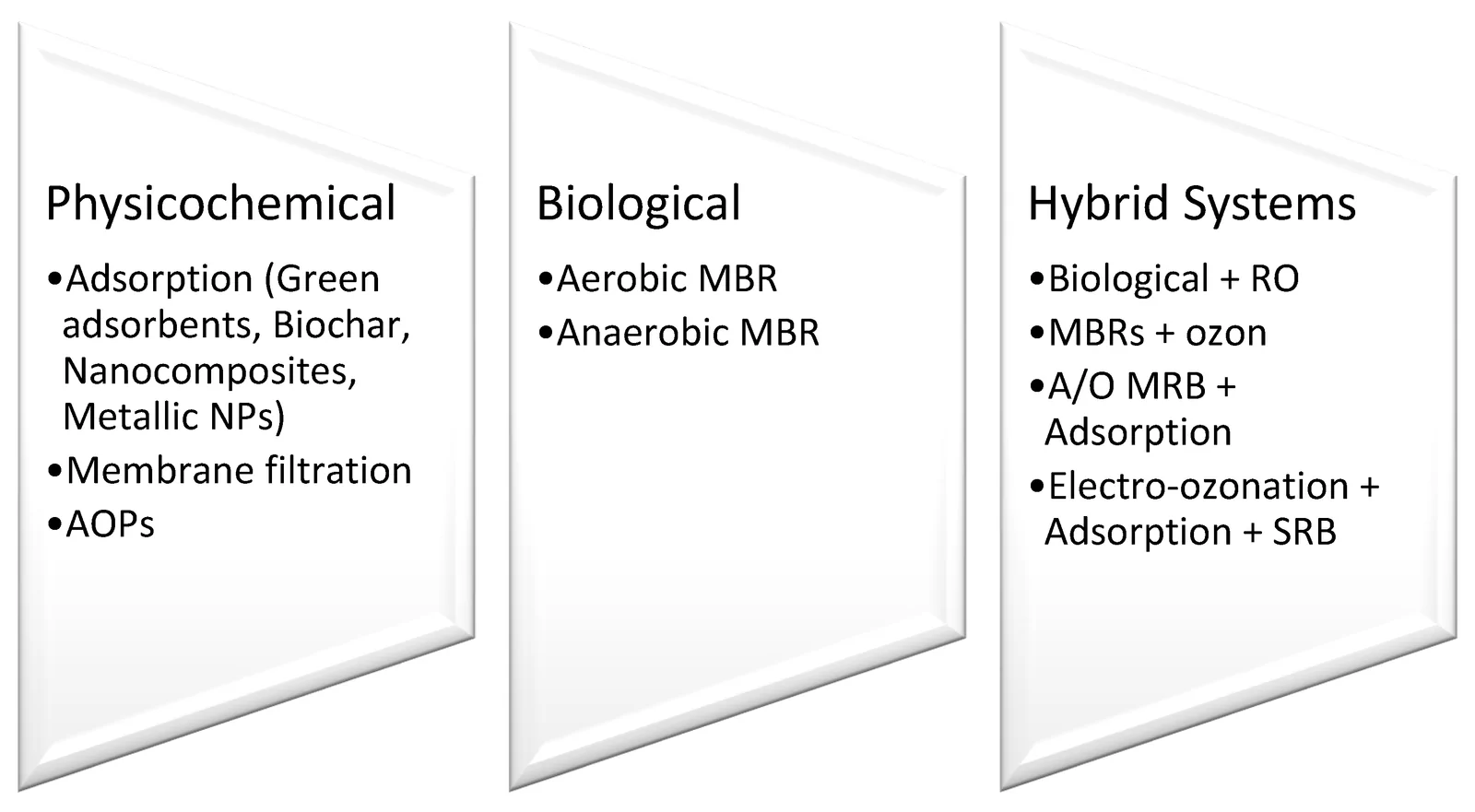
The main methods for the treatment of landfill leachate.

Physicochemical (adsorption, membrane filtration, and advanced oxidation process), biological processes, or a combination of biological and physicochemical treatment are the well-known techniques reported to treat complex landfill leachate (Figure 4) [4,6,7,8,9,56,63,84,85,86,87,88].

Table 3 summarizes several studies on landfill leachate treated by physicochemical or biological methods, highlighting leachate characteristics and the performance in terms of pollutants removal.

#### 3.3.1. Physicochemical Treatment

Adsorption is a widely used physicochemical technique for the removal of residual contaminants. An ideal adsorbent should possess abundant functional groups (–OH, –C=O), well-developed microporosity, and a large specific surface area (SSA) to effectively remove pollutants from landfill leachate. In addition, it should be prepared at a low cost and function as an environmentally friendly alternative to commercial AC.

According to Table 3, the following natural adsorbents have been successfully integrated for the treatment of landfill leachate: crosslinked-protonated chitosan (CHT-CLP) [4], nano-graphene oxide (nGO), carboxylate-functionalized multi walled carbon nanotubes (MWCNT-COOH), iron doped-carboxylate-functionalized multiwalled carbon nanotubes (MWCNT-COOH-Fe_3_O_4_) [6], green mussel (*Perna viridis*) [7], oat husks [8], rice husk ash [92], peat [39], biochar [54,56,60,83,93], Fe NPs [59], and magnetic nanocomposites [91].

Using a low-cost biochar produced through self-sustained carbonization of wood waste, Samsudin et al. [83] achieved excellent removal efficiencies for COD (73%), NH_4_^+^-N (70%), TKN (97%), and Cr (99.8%), Cu (94.4%), Fe (98.5%), and Ni (99.4%), with the treated effluent fulfilled the Malaysian discharge standards (suspended solids of 50 mg/L, color of 100 Pt–Co, COD of 400 mg/L, and a BOD_5_ concentration of 20 mg/L). The wood waste biochar prepared from feedstock with a moisture content of 40–42% (WCBC-40) exhibited an SSA of 232.1 m^2^/g, an average pore size of 15.4 nm, an ash content of 3.8%, a biochar yield of 20%, and an O/C atomic ratio of 0.3, resulting in superior contaminant removal compared with conventional AC.

A good approach to achieve the best removals of COD and color is to use activated biochar. Thus, the 100% impregnated oat hull with H_3_PO_4_, followed by pyrolysis at 500 °C, showed an SSA of 3880 m^2^/g. After a contact time of 3 h with real leachate, a total removal of color and COD of 90 ± 0.5% was achieved [8].

#### 3.3.2. Biological and Integrated Treatments

The biological and hybrid treatment technologies that have been evaluated include anaerobic MBRs [63], sequencing batch reactor (SBR) coupled with powdered ZELIAC (PZ) adsorbent [9], A/O MBR integrated with a tertiary adsorption unit [4], biological pre-treatment followed by reverse osmosis (RO) [61], AOP and post-MBR [86], and electro-ozonation combined with a BAZLSC composite adsorbent augmented sequencing batch reactor (SBR) process (PB-SBR) [23].

Aerobic processes are suitable for the treatment of young landfill leachate due to their effective removal of biodegradable organic matter.

Biological treatment combined with membrane filtration or adsorbents is a promising approach for the treatment of mature landfill leachate. Thus, a membrane bioreactor (MBR) can be considered a conventional activated sludge system integrated with membrane filtration. Lindamulla et al. [63] evaluated the treatment of mature landfill leachate in a tropical climate (Sri Lanka) using different MBR configurations. The highest removal efficiencies of BOD_5_, COD, and TN were recorded at a sludge retention time (SRT) of 60 days, and the leachate had a hydraulic retention time (HRT) of one day.

Mojiri et al. [9] investigated the sequencing batch reactor (SBR) combined with powdered ZELIAC (PZ) adsorbent composed of Portland cement, limestone, rice husk ash, activated carbon, and zeolite for treating municipal sanitary landfill leachate mixed with wastewater and activated sludge. PZ was characterized by a multipoint BET of 132.8 m^2^/g, a size of 75–150 μm, and an average pore diameter of 2.384 nm, working both as an adsorbent and ion exchanger at the same time, which improves the performance of the SBR process.

Dira et al. [61] studied biological pre-treatment coupled with reverse osmosis (RO) as a new strategy for treating landfill leachate. The authors reported above 99% removal efficiencies for COD, BOD_5_, TP, and NH_4_^+^-N, as well as a drastic reduction in leachate pollution index (LPI) from 80 to 100 to ˂10. The major challenges of this study are the additional treatment required for the removal of TKN and to meet regulatory compliance, as well as the management of the RO concentrate.

In contrast, anaerobic processes are better suited for treating high-strength leachate because they can handle higher organic loading rates and enable resource recovery through biogas production. Wang et al. [60] developed an S,N-doped multifunctional biochar with a high specific surface area of 330.56 m^2^ g^−1^, which acted simultaneously as an adsorbent, catalyst, and microbial support material during the anaerobic digestion of landfill leachate. The modified biochar enhanced the removal of aromatic compounds through combined adsorption and biodegradation, reduced toxicity and inhibitory effects, and provided a favorable surface for microbial colonization. As a result, the CH_4_ production increased during the stabilization phase, reaching a specific methanogenic activity (SMA) of 50.07% on day 14.

To take advantage of the strengths of anaerobic and aerobic processes, combined anaerobic/aerobic (A/O) systems have been widely adopted [4,14]. These systems achieve a superior treatment performance by the simultaneous removal of COD and NH_4_^+^-N, resulting in higher overall removal efficiencies than either process alone. For instance, Wijerathna et al. [4] investigated an Anoxic-Oxic Membrane Bioreactor (A/O MBR) process integrated with the CHT-CLP adsorbent, characterized by a SSA of 38.42 m^2^/g (BET) and a pH_ZPC_ value of 4.8 for the removal of COD, BOD_5_, NO_2_^−^, and color. Using Response Surface Methodology (RSM), the integrated process achieved nearly 100% removal efficiency for NO_2_^−^ and color. However, the treated effluent still exceeded the discharge limits for BOD_5_ (30 mg/L) and COD (250 mg/L) values.

A powdered BAZLSC adsorbent was developed as a reasonable-cost material composed of crushed bentonite, zeolite, activated carbon, cockle shell, limestone, and Portland cement [23]. It is characterized by a BET surface area of 288 m^2^/g and a micropore area of 61.9 m^2^/g. BAZLSC used in combination with electro-ozonation and activated sludge led to the sequencing batch reactor (SBR) step. O_3_ can improve the efficiency of biological treatment by decreasing substrate toxicity and oxidizing complex organic compounds into simpler, more biodegradable molecules.

#### 3.3.3. Comparative Performance of Different Technologies for the Treatment of Landfill Leachate

**Figure 5:**
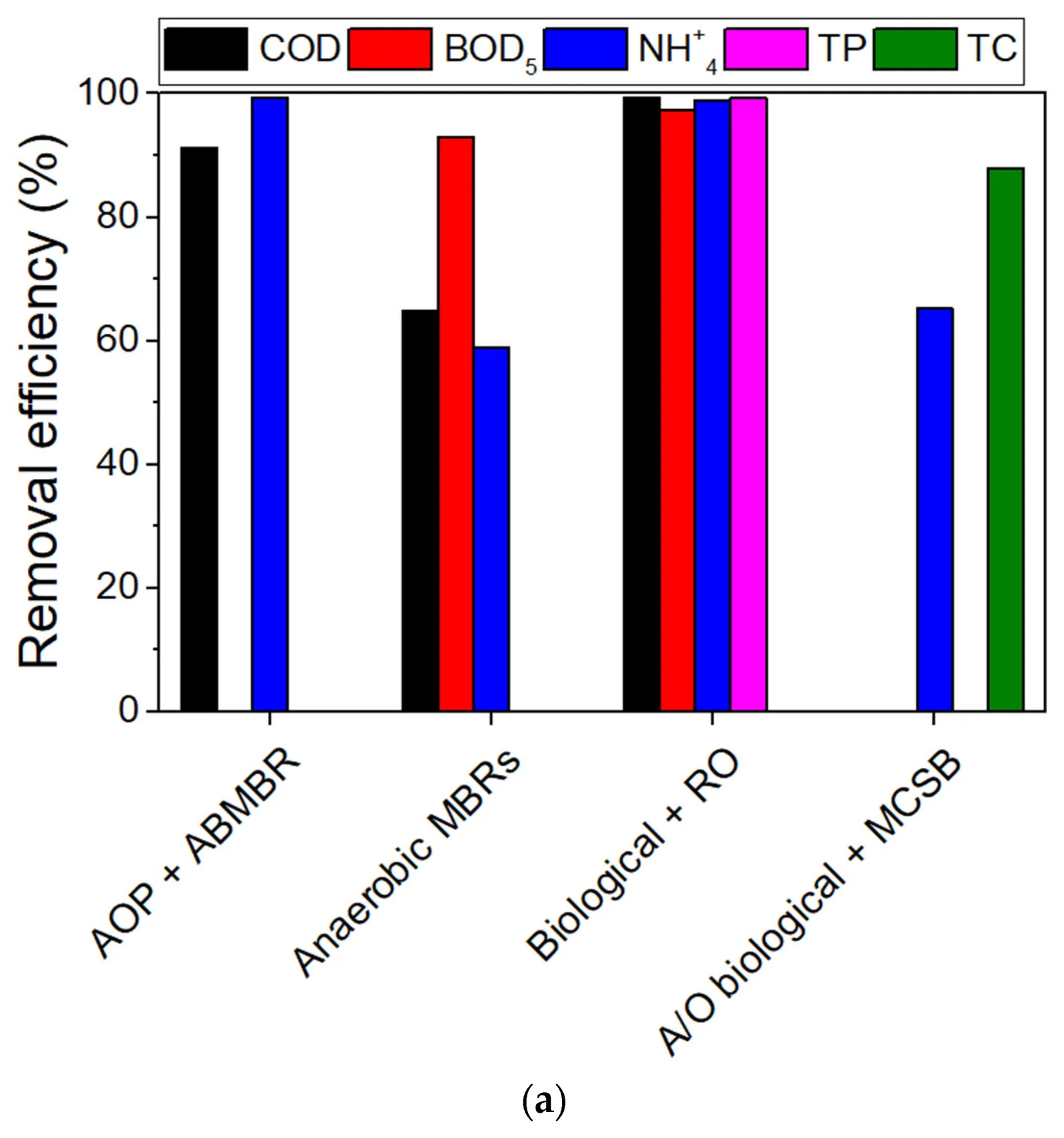

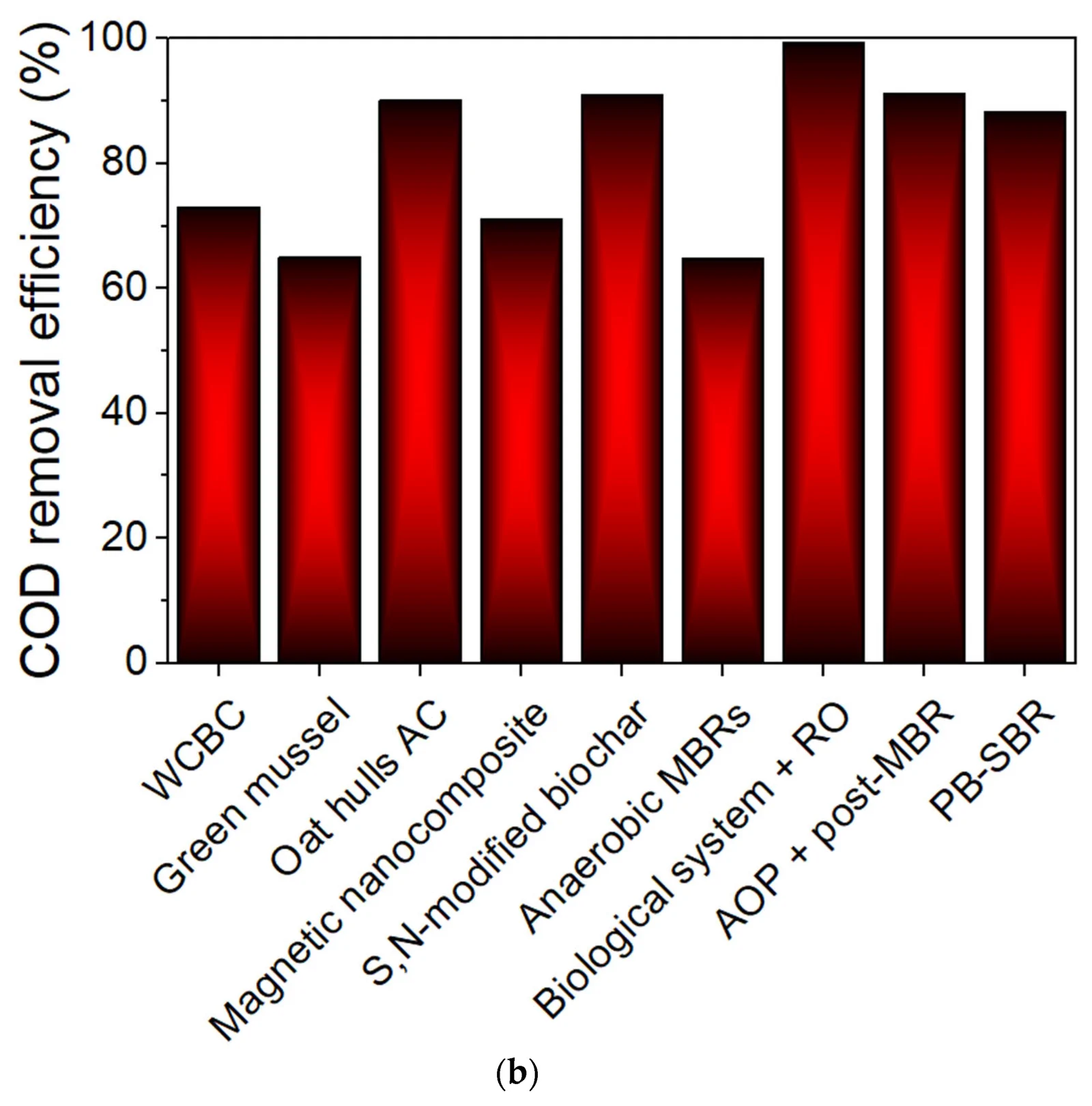
The most efficient technologies for removal of COD, BOD_5_, TC, NH_4_^+^, and TP (**a**) and the profile of COD removal efficiency among all investigated technologies (**b**).

Figure 5a,b shows a comparison of COD, BOD_5_, NH_4_^+^, TP, and TC removal by different leachate treatment processes.

According to Table 3, it can be observed that hybrid treatment is more efficient for the removal of COD, NH_4_, and tetracycline antibiotics.

Figure 5a compares the removal efficiencies of several biological and hybrid treatment processes for landfill leachate. Among the evaluated systems, the biological treatment combined with reverse osmosis (biological + RO) [61] exhibited the broadest treatment performance, achieving high removal efficiencies of 99.31% for COD, 97.4% for BOD_5_, > 99% for NH_4_^+^, and 99.27% for TP. Advanced oxidation processes (AOPs) coupled with an anaerobic baffled membrane bioreactor (ABMBR) [86] also achieved high removal efficiency of 91.2% of COD and 99.4% for NH_4_^+^. In contrast, anaerobic membrane bioreactors (MBRs) [63] showed the removal of organic matter (COD) of 64.8%. The anaerobic/aerobic (A/O) biological system supplemented with magnetic coconut shell biochar (MCSB) [14] was specifically evaluated for tetracycline (TC) and ammonium removal, achieving removal efficiencies of approximately 90% for TC and 65% for NH_4_^+^.

Figure 5b illustrates the COD removal efficiencies reported for selected adsorbents and treatment technologies based on the literature summarized in Table 3. The highest COD removal efficiency was achieved by the biological treatment combined with reverse osmosis (Biological system + RO) [61], reaching approximately 99%, demonstrating the superior performance of membrane-based polishing processes. Other advanced treatment systems, including AOP coupled with a post-membrane bioreactor (AOP + post-MBR) [86], S,N-modified biochar [60], and the PB-SBR process [23], also exhibited excellent COD removal efficiencies of approximately 90–91%. In comparison, oat hulls activated carbon (AC) achieved about 89% COD removal [8], indicating that low-cost bio-based adsorbents can provide a comparable efficiency to more complex hybrid systems. Moderate removal efficiencies (approximately 70–73%) were obtained using biochar from wood waste (WCBC) [83] and a magnetic GO–Fe_3_O_4_/WO_3_ nanocomposite [91], whereas green mussel [7] and anaerobic membrane bioreactors (MBRs) [63] showed the lowest COD removal efficiencies (approximately 65%).

However, the successful long-term operation of membrane-based systems depends on the effective control of fouling. In the management of landfill leachate using membrane technology, an increase in the pH to alkaline values promotes the formation of complexes between Mg^2+^ and Ca^2+^ and humic substances and polysaccharides, thereby contributing to membrane fouling [84]. The proposed management strategies for reverse osmosis (RO) concentrates include evaporation and crystallization for the recovery of salts and inert organic matter, together with the reuse of the concentrate as a leaching agent in biomass or waste pyrolysis/gasification processes [61].

**Figure 6:**
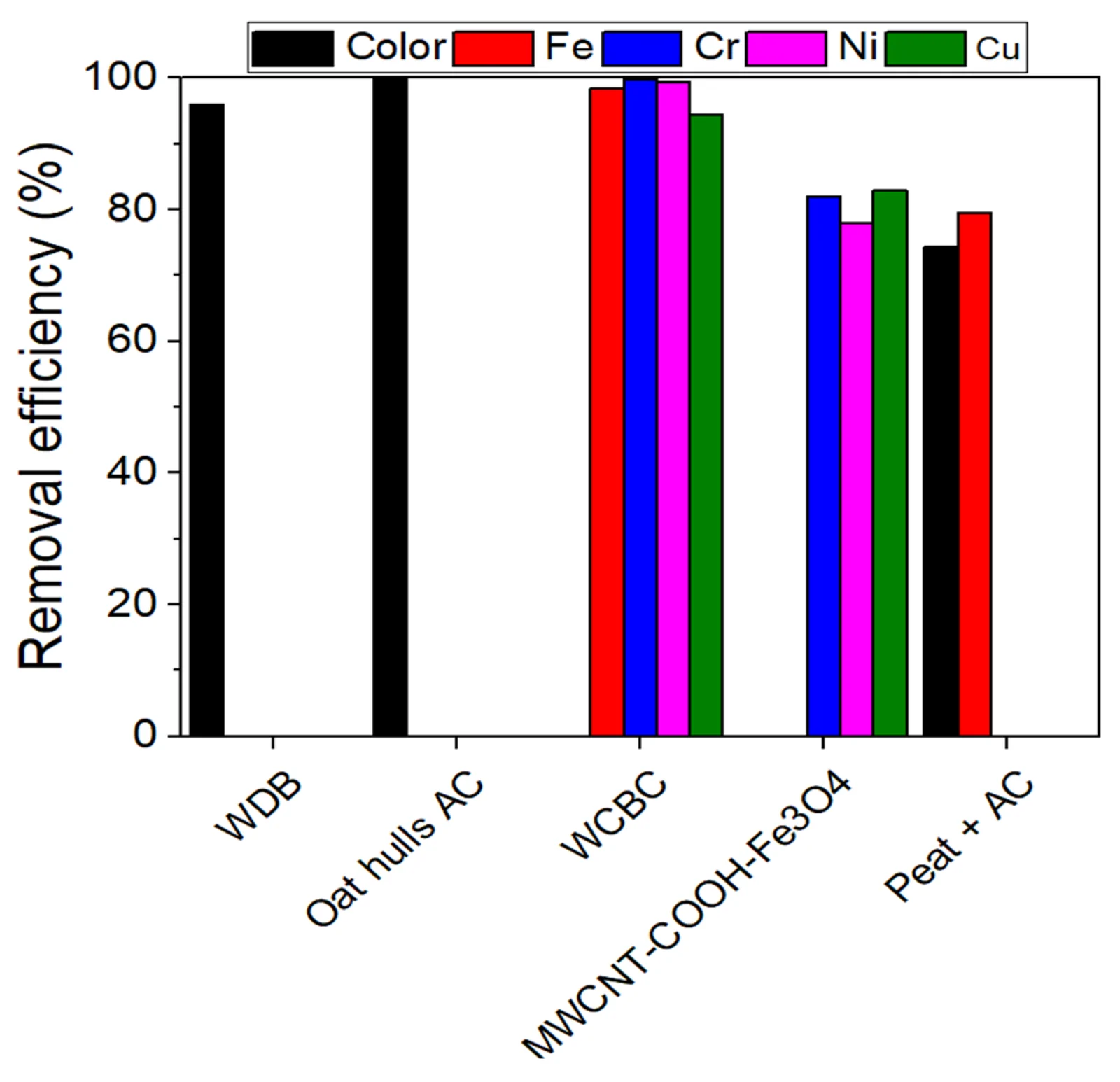
Effect of adsorbent types on discoloration and removal of some heavy metals from leachate.

Figure 6 compares the removal efficiencies (%) of different adsorbents for color and Fe, Cr, Ni, and Cu ions from landfill leachate.

The selection of an adsorbent should be based on the target pollutants. The highest color removal was reported for the oat hulls AC (100%) [8], followed by WDB (~95%) [90], while the peat mixed with AC indicated a modest adsorption of colored organic compounds of 74.4% [39]. Modified biochar (WCBC) exhibited the best overall performance for heavy metal removal, with removal efficiencies of approximately 98% for Fe, 100% for Cr, 99% for Ni, and 94% for Cu [83]. This suggests that WCBC possesses abundant active adsorption sites and a high affinity for dissolved metal ions.

MWCNT-COOH-Fe_3_O_4_ exhibited moderately high removal efficiencies, removing approximately 82% of Cr, 78% of Ni, and 83% of Cu, demonstrating that magnetic functionalization enhances heavy metal adsorption but is less effective than WCBC [14]. Similarly, peat mixed with AC retained 79% Fe [39].

## 4. Advances in Sludge-Based Adsorbents for the Treatment of Landfill Leachate

### 4.1. Sewage Sludge-Based Adsorbents

SBAs have attracted increasing attention as sustainable materials for landfill leachate treatment because they promote the valorization of sewage sludge and reduce the dependence on expensive commercial adsorbents. Although the conversion of sewage sludge into adsorbents has been increasingly investigated [26,94,95], its application in the treatment of landfill leachate remains an emerging research area that requires comprehensive evaluation.

Table 4 summarizes the recent studies on the application of sewage sludge-derived materials as multifunctional adsorbents, catalysts, and microbial carriers for the removal and degradation of recalcitrant contaminants from landfill leachate.

#### 4.1.1. Strategies for Improving the Adsorption of SBAs

Among the available thermochemical conversion processes, pyrolysis is the most widely used to transform sewage sludge into biochar, which has diverse applications including soil amendment, biogas, adsorbent, and electrochemical capacitors [60,99,102,103,104,105,106]. The estimated cost of pyrolysis for treated sludge ranged from USD 4000 to USD 7000 per ton [107].

However, the adsorption performance of sludge-derived biochar is highly dependent on its physicochemical characteristics, which are strongly influenced by pyrolysis temperature and residence time.

The yield of biochar decreases with increasing of pyrolyzing temperature [108]. Zhao et al. [109] reported that a pyrolysis temperature of 600 °C was optimal for preparing SBAs, whose capacity for removing Pb(II) from landfill leachate was strongly influenced by their surface properties. At this optimum temperature, the SBAs showed a pH of 8.9, an ash content of 75.5%, and cation exchange capacity (CEC) of 4 cmol/kg. The prepared SBAs exhibited a surface area of 22.4 m^2^/g and a maximum Pb(II) adsorption capacity of 132 mg/g.

Tawfik et al. [110] investigated the application of hardwood biochar as an additive in an anaerobic baffled reactor for the treatment of landfill leachate containing 3,5-dimethylphenol. After biological treatment, the excess sludge was subjected to pyrolysis at 550 °C to produce a value-added biochar. The recovered biochar contained abundant oxygen-containing functional groups, which enabled the adsorption of the remaining organic pollutants and provided an additional polishing step for the treated effluent. This approach simultaneously improved landfill leachate treatment efficiency while promoting sludge valorization and resource recovery within a circular economy framework [110].

However, unmodified biochar typically exhibits poor adsorption performance due to the limited availability of functional adsorption sites. For instance, adsorbents based on green mussel, showing a particle size range from 2.00 mm to 3.35 mm and an SSA of 95.95 m^2^/g, showed removal efficiencies of COD and NH_3_-N of 65% and 45%, respectively [7]. This limitation is particularly pronounced for anionic species, as the predominantly negatively charged biochar surface electrostatically repels negatively charged ions [111].

An approach to enhance the surface area and porosity of sludge-derived biochar was proposed by Giroto et al. [112]. The authors produced AC from industrial sewage sludge collected from a WWTP through H_3_PO_4_ activation followed by pyrolysis at 450 °C for 1 h. At impregnation ratios of the industrial sewage sludge to H_3_PO_4_ of 1:1 and 1:3, with activation at a temperature of 140 °C for 3 h, the resulting AC exhibited BET surface areas of 60 and 820 m^2^/g, respectively, and average pore diameters of 9.81 and 3.31 nm, respectively. Furthermore, H_3_PO_4_ was recovered by collecting the wash water after activation and reusing it in subsequent activation cycles, demonstrating the feasibility of a circular and resource-efficient activation process.

In addition to conventional chemical activation, alternative and more sustainable activation strategies have also been explored to enhance the physicochemical properties of sludge-derived biochar. For instance, Li et al. [105] achieved a biochar with a high surface area of 480.3 m^2^/g using seawater as a bioactivating agent. In their proof-of-concept study, sludge particles impregnated with seawater were pyrolyzed in a tubular furnace under a constant nitrogen atmosphere at 750 °C, with a heating rate of 10 °C/min and a residence time of 1 h, followed by washing with 6 N HCl.

Beyond metal removal, AGS also exhibits a strong capacity for the immobilization of other contaminants through adsorption mechanisms. Peng et al. [113] demonstrated that model nanoparticles (NPs) presented in WWTP could be effectively captured by the AGS system, with approximately 85% of the adsorption occurring within the first 30 min. At an influent NPs concentration of 143.79 μg/L, 94.60–98.67% of the NPs were adsorbed onto the sludge matrix during each Nereda^®^ cycle, while desorption efficiencies remained below 5.0% during both the plug-flow feeding and aeration phases.

The integration of vegetation treatment with activated sludge processes can modify sludge composition. Further, this approach improves landfill leachate treatment by enriching microbial communities responsible for nitrogen removal and organic pollutant degradation, as confirmed by predicted metabolic function analysis [114]. Wdowczyk et al. [56] proposed an integrated biological system operated in laboratory-scale sequential batch reactors (SBRs), in which the activated sludge, aquatic plants, and adsorbent media (biochar and/or zeolite) worked together to improve the treatment of mature landfill leachate. Three plant species (*Typha minima* (dwarf bulrush), *Lythrum salicaria* L. (purple loosestrife), and *Iris pseudacorus* (yellow flag)) were selected due to their ability to remove nutrients and immobilize heavy metals. The integrated system demonstrated high treatment performance, with 100% TP removal and a final COD concentration of 10.9 mg O_2_/L, while maintaining the long-term stability of the adsorbents, offering a cost-effective and sustainable approach for landfill leachate management.

Beyond adsorption of contaminants, biochar provides a porous habitat for biofilm development, promotes extracellular electron transfer, and enhances microbial metabolic activity, thereby improving the removal of refractory organic compounds, heavy metals, and nitrogenous pollutants. Consequently, the synergistic integration of biochar with biological treatment systems mitigates the limitations of conventionally activated sludge processes under the toxic and complex conditions of landfill leachate, providing an effective and sustainable strategy for enhanced contaminant removal [13].

#### 4.1.2. Performance of SBAs

**Figure 7:**
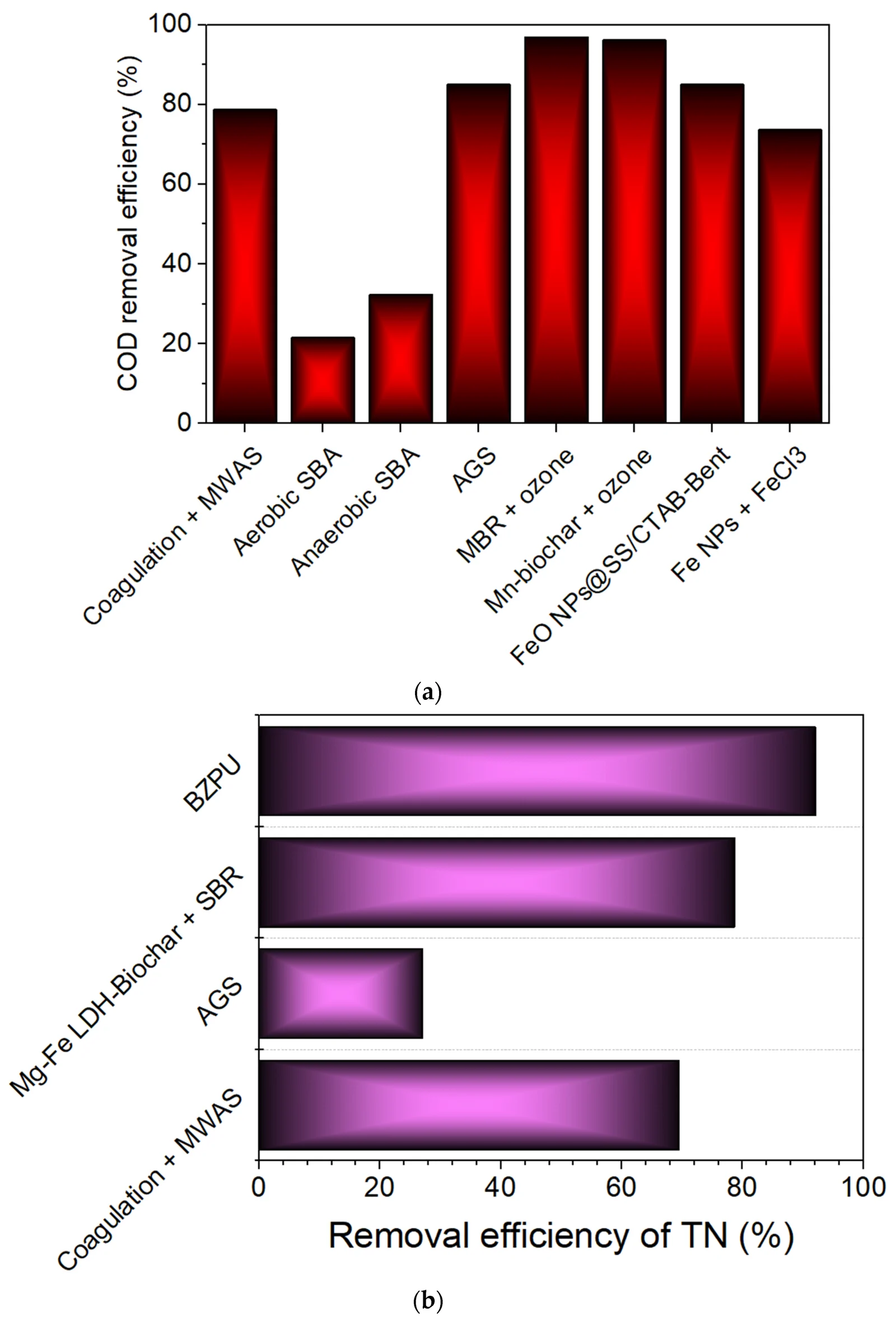
Performance of SBAs for treating contaminated landfill leachate. COD removal efficiencies (**a**) and TN removal efficiencies (**b**).

Figure 7a,b compares the efficiency of different technologies, including SBAs, to remove COD and TN from landfill leachate.

Among the evaluated technologies, membrane bioreactor (MBR) coupled with ozonation and magnetic biochar combined with ozonation (Mn-biochar + ozone) achieved the highest degradation of refractory organic matter, both reaching removal efficiencies above 96% [99,100]. This performance could be explained by the impregnation of the biochar with Mn, which increased the abundance of the reducing functional groups (C–O and C–C), and promoted O_3_ decomposition and the generation of reactive oxygen species (ROS), including **^·^**OH, **^·^**O_2_^−^, **^·^**O_2_^2−^, and ^1^O_2_. These ROS enhanced the oxidation and degradation of organic pollutants, while denitrifying bacteria in the activated sludge synergistically promoted the removal of nitrogenous compounds [100]. In the case of a membrane bioreactor (MBR) coupled with ozonation and magnetic biochar, the biochar was produced by carbonizing dried in a tube furnace at 800 °C [99]. By coupling catalytic ozonation with Mn-loaded biochar (particle size of 2500 μm) derived from residual sludge, a gradual decrease in NH_4_^+^-N concentration was accompanied by a corresponding increase in NO_3_^−^-N (143.96 mg/L). This trend was attributed to the oxidation pathway of ammonium, where NH_4_^+^-N was first converted to NO_2_^−^-N and subsequently oxidized to NO_3_^−^-N under catalytic ozonation conditions [100].

FeO NPs supported on cetyltrimethylammonium bromide-modified bentonite (FeO NPs@CS/CTAB-Bentonite) and aerobic granular sludge (AGS) also demonstrated high treatment performance, each achieving COD removals of approximately 84–85% [55,97].

Sewage sludge coated with FeO NPs and organically modified bentonite is a low-cost composite adsorbent with a surface area of 37.11 m^2^/g, which led to an increase in the number of active adsorption sites and improved with ≥85% removal of Cd(II), COD, and NH_3_-N compared with unmodified sludge [55]. Aerobic granular sludge (AGS) technology provides a promising route for generating sludge-derived carbon materials with enhanced structural stability and microbial-derived extracellular polymeric substances (EPS), which can contribute to the pore formation and surface functionality after carbonization [97]. In addition, the AGS maintained its structural and functional stability during the regenerative adsorption–desorption cycle, when 47% of the Cu, Pb, Fe, Mn, and Zn uptake occurred via bioaccumulation within the biomass [97]. A 27% removal of TN was reported [87].

Similarly, coagulation followed by adsorption using modified waste activated sludge (MWAS) removed approximately 78% of COD, indicating that adsorption-based approaches can effectively reduce the organic load [96]. The coagulation stage was carried out using poly-aluminum chloride (PACl), followed by adsorption onto MWAS. The waste activated sludge (characterized by a pH of 7.4–7.6, a total suspended solids concentration of 2.9 g/L, and sludge age of 12 days) was modified by acid treatment using 1 M HCl (1:1 *w*/*v*), for 24 h at room temperature. The maximum adsorption capacity predicted by the Langmuir model reached 103.09 mg COD/g [96]. Also, a moderate TN removal efficiency of approximately 69% was reported [96].

In contrast, Fe NPs combined with FeCl_3_ achieved a moderate COD removal of approximately 73% [101], whereas anaerobic sludge adsorption (SBA) and aerobic SBA exhibited substantially lower efficiencies, with COD removals of approximately 32% and 21%, respectively [29]. The biosynthesized spherical Fe NPs with diameters ranging from 13 to 150 nm were achieved by treating the supernatant obtained from sludge generated during the coagulation-flocculation process of landfill leachate with H_2_SO_4_ and black tea extract [101]. Pearson’s correlation analysis indicated that the COD removal was favored at lower pH values, while higher SSA was positively correlated with the improved removal of COD, DOC, and color [29].

A similar trend was observed for TN removal, where hybrid technologies outperformed conventional biological and physicochemical processes. BZPU, a combination of sludge-derived biochar with nano zero-valent iron (nZVI) and a modified polyurethane biocarrier, achieved the highest TN removal efficiency (approximately 92%) [98]. In this approach, nZVI complemented the functions of biochar by further enhancing extracellular electron transfer and creating favorable redox conditions for microbial activity. As a result, the modified biocarrier promoted the simultaneous partial nitrification–anammox and denitrification processes within the biofilm. However, this approach failed to remove low-biodegradable COD.

The Mg–Fe layered double hydroxide (LDH)-biochar coupled with a sequencing batch reactor (SBR) removed approximately 79% of TN [13]. This biocomposite exhibited an SSA of 208.115 m^2^/g and acted as both adsorbent and catalyst for the treatment of landfill leachate loaded with bisphenol A (BPA), PO_4_^3−^-P, and humic acid, achieving removal efficiencies of 96.8%, 94.55%, and 94.34%, respectively [13].

### 4.2. Adsorption Mechanism of Sludge-Based Adsorbents

The Langmuir model assumes monolayer adsorption on a homogeneous surface with uniform adsorption sites, whereas the Freundlich model describes multilayer adsorption on heterogeneous surfaces with variable adsorption energies.

The adsorption equilibrium for the removal of pollutants from landfill leachate in the presence of green mussel adsorbent was well described by the Langmuir model for COD (R^2^ = 0.9979) and the Freundlich model for NH_3_-N (R^2^ = 0.9938) [7].

Langmuir model (R^2^ = 0.979) best described the color and COD removed by the monolayer adsorption in the case of the CHT-CLP and magnetic GO–Fe_3_O_4_/WO_3_ nanocomposite adsorbents [4,91].

Ab Ghani et al. [115] investigated the adsorption mechanisms and kinetics of COD, DOC, and color removal from a stabilized landfill leachate in Malaysia using AC derived from banana stem, iron oxide nanocomposite (IOAC), and iron oxide nanoparticles (IONPs) adsorption systems in a continuous fixed bed column. Their findings revealed maximum COD adsorption capacities of 476.19 mg/g, 526.32 mg/g, and 69.44 mg/g for AC, IOAC, and IONPs, respectively. The adsorption data for all treatments showed a good fit with the Langmuir isotherm model, and the adsorption kinetics were best described by the pseudo-second-order model, suggesting that adsorption was likely controlled by the availability of active sorption sites and involved interactions between the pollutants and functional groups on the adsorbent surface.

Similarly, the experimental data of the nZVI adsorbent fitted well to the Langmuir isotherm model and pseudo-second-order kinetic model (R^2^ > 0.9), providing higher removal efficiencies for dissolved organic matter and nitrogen species [59].

Also, the Langmuir adsorption isotherm better described the removal of Mn (R^2^ = 0.9743), since Freundlich adsorption isotherms characterize the removal of Fe (R^2^ = 0.9219), Ni (R^2^ = 0.9292), and Cd (R^2^ = 0.9085) in the case of powdered ZELIAC (PZ) adsorbent [9].

In addition, the adsorption kinetics of AC derived from oat hulls, impregnated with H_3_PO_4_, and subsequently pyrolyzed, as well as those of a CHT-CLP adsorbent integrated into an A/O MBR, were best described by the pseudo-second-order models, with R^2^ = 0.995 and k_2_ = 0.004 g mg^−1^ min^−1^ [8] and R^2^ = 0.9861 and k_2_ = 0.00068 g mg^−1^ min^−1^ [4], respectively. A similar kinetic model was reported for the magnetic GO–Fe_3_O_4_/WO_3_ nanocomposite used as an adsorbent, which demonstrated an excellent adsorption capacity of COD of 3561.83 mg/g [91]. These results suggest that chemisorption was the rate-limiting step in both systems.

Wang et al. [111] described the mechanism of phosphate removal by a lanthanum (La) loaded fabricated biochar from waste sludge. The maximum adsorption capacity of ~94.4 mg P/g was achieved at a pyrolysis temperature of 500 °C and pH 3. This biochar surface exhibited a pH-dependent charge characteristic (pH_pzc_ ≈ 6.2), with protonated surfaces below the pH_pzc_ promoting phosphate adsorption via electrostatic attraction, although this outer-sphere complexation mechanism may be weakened by competing anions. The phosphate adsorption kinetics were best described by the pseudo-second-order model (R^2^ = 0.9762–0.9876), indicating that chemisorption governed the adsorption process through electron sharing or exchange between the adsorbent and phosphate ions. Adsorption was controlled by both film diffusion and intraparticle diffusion, with the external mass transfer occurring at a faster rate. The Langmuir model accurately described the adsorption isotherms, yielding maximum adsorption capacities of 114.5 mg P/g at pH 3 and 87.4 mg P/g at pH 6 (at a temperature of 23 °C).

The enhanced adsorption performance of a low-cost biochar produced through self-sustained carbonization of wood waste for the removal of COD (73%), NH_4_^+^-N (70%), TKN (97%), and Cr (99.8%), Cu (94.4%), Fe (98.5%), and Ni (99%) was attributed to the combined effects of physisorption, electrostatic interactions, and hydrogen bonding involving the carboxyl (–COOH), hydroxyl (–OH), and alcohol (R–OH) functional groups, together with oxygen-containing functional groups generated during the carbonization process [83].

**Figure 8:**
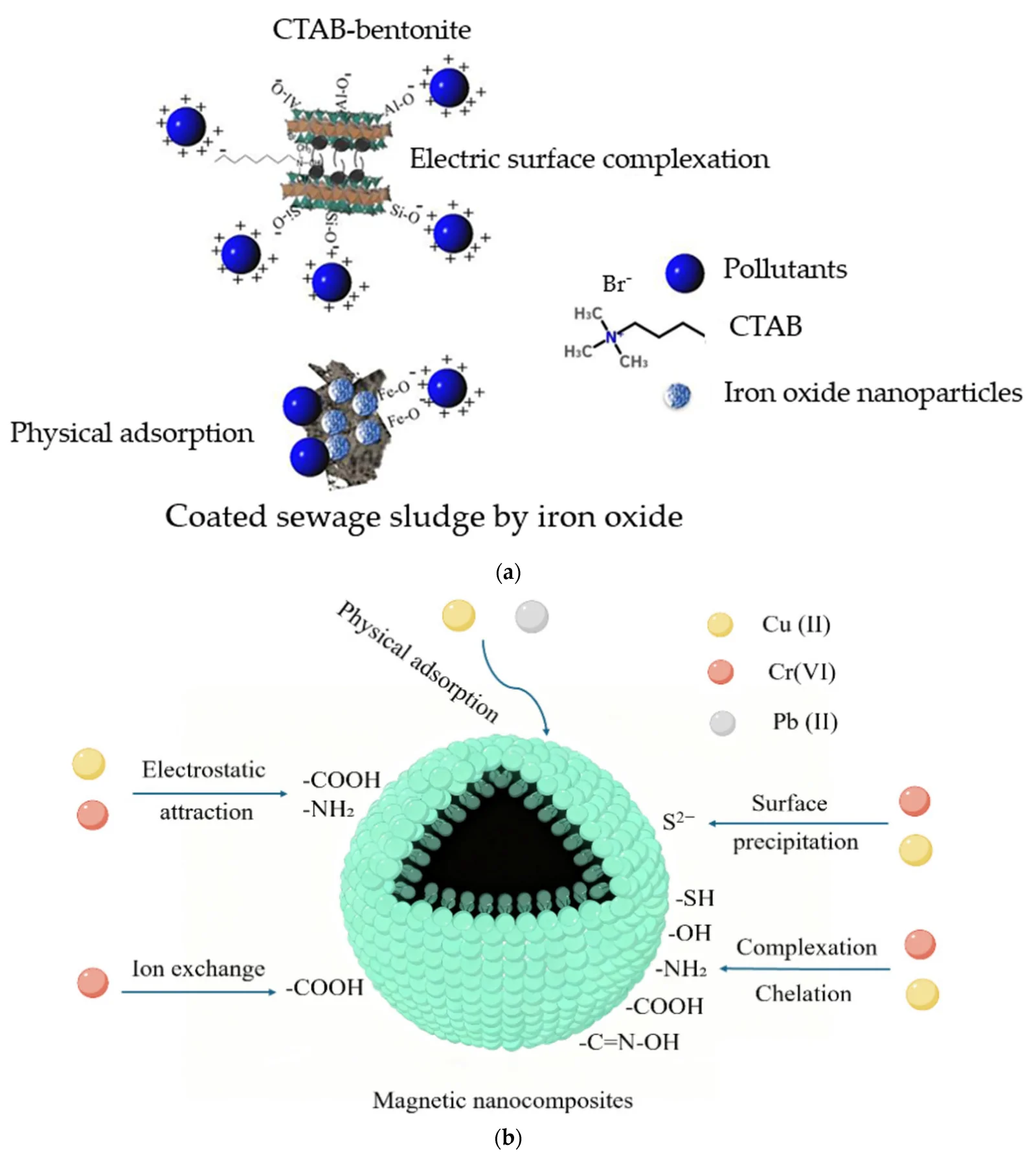
Illustration of the adsorption mechanism of COD, NH_3_-N, and Cd(II) in the presence of coated sewage sludge with FeO NPs and combining it with organically modified bentonite (**a**) [55] (Open Access), and adsorption mechanism of heavy metals in the presence of magnetic nanocomposites (**b**) [116] (Open Access).

Ahmed et al. [55] explained the efficient removal of pollutants from synthetic landfill leachates by the cetyltrimethylammonium bromide (CTAB)-modified bentonite/iron oxide composite—Figure 8a.

According to the authors [55], the removal of COD, NH_3_-N, and Cd(II) occurred through two primary mechanisms: electrostatic surface complexation and physical adsorption. The positively charged CTAB molecules modified the clay surface, increasing its affinity for organic contaminants and promoting electrostatic interactions between the adsorbent surface and dissolved pollutants. Meanwhile, the iron oxide-coated sewage sludge contributed to the pollutant removal through physisorption and surface interactions involving Fe-O functional groups (Figure 8a). The synergistic action of these mechanisms resulted in excellent adsorption performance, with equilibrium adsorption capacities (Q_e_) of 0.556 mg/g for Cd(II), 102 mg/g for COD, and 10.42 mg/g for NH_3_-N, demonstrating the efficiency of the composite.

The high removal mechanism of Cr, Cu, Fe, and Ni (>94%) in the presence of wood waste biochar (WCBC) was explained by the formation of oxygen-containing functional groups on the biochar structure, due to the higher carbonization temperature, which interact with positively charged heavy metal ions. The alkalinity of biochar suggests the ability of the biochar to neutralize the acidity of the leachate [83].

As illustrated in Figure 8b, the removal of Cu(II), Cr(VI), and Pb(II) was governed by multiple synergistic mechanisms, including electrostatic attraction, ion exchange, physical adsorption, surface precipitation, complexation, and chelation [116]. Functional groups such as –COOH, –NH_2_, –OH, –SH, and =N–OH on the adsorbent surface provided abundant active sites for binding metal ions through electrostatic interactions and coordination with surface functional groups. In addition, S^2−^ species promoted the surface precipitation of Cu(II), Cr(VI), and Pb(II) ions, further enhancing their removal.

### 4.3. Environmental Risks of Heavy Metals Leached from Biochar

A potential limitation of sewage sludge-derived biochar is the presence of heavy metals originating from the feedstock [117,118].

The Sewage Sludge Directive 86/278/EEC establishes the limit values for concentrations of heavy metals from sludge for use in agriculture and soil—Table 5 [69].

Morales et al. [108] conducted an LCA to evaluate the toxicological impacts of raw sewage sludge from six Norwegian WWTPs treated by pyrolysis. Biochar was produced at temperatures ranging from 500 to 800 °C with a residence time of 2 h. Twelve heavy metals, As, Ba, Cd, Co, Cr, Cu, Mo, Ni, Pb, Sr, V, and Zn, were quantified in the raw sewage sludge. The study found that, in the absence of anaerobic digestion, pyrolysis increased the concentration of heavy metals in the resulting biochar by approximately 70% and promoted the release of Zn into the atmosphere. Heavy metals were identified as the dominant pollutants affecting both human health (>93% of the total impact) and freshwater ecotoxicity potential (approximately 100%). The major contributors to human health impacts through emissions to air and water were ranked as follows: Zn > Pb > Cd > As > Ba. Zn was the primary contributor, accounting for 38–81% of the total human health impact. For freshwater ecotoxicity, Zn also dominated, contributing 55–59% of the total impact, followed by Sr and Cu, each contributing up to 17%.

The accumulation of heavy metals in biochar also increased the potential risk of soil contamination. However, the long-term stability of heavy metals in sewage sludge-derived biochar improved when pyrolysis was conducted at temperatures above 600 °C. These higher temperatures led to a decrease in the leachability of heavy metals because they were transformed into more stable chemical species [108,119]. At higher temperatures, the biochar also becomes increasingly aromatic and graphitic in structure. The increase in aromaticity reduces the abundance of oxygen-containing functional groups on the biochar surface. The reduced toxicity and the potential for heavy metals leaching into groundwater are due to the stability and less mobility of chemical species.

Kujawska [118] assessed the mobility and environmental risks associated with heavy metals in biochar. The results showed that the concentrations of Cr, Ni, Cu, Zn, Pb, and Cd did not exceed the limits established by the International Biochar Initiative (IBI). Ecotoxicity assessment of the biochar, conducted in accordance with the EN 12457-2:2006 standard [119], indicated negligible leaching of heavy metals. However, the geo-accumulation index (GAI) identified Cd as posing the highest potential risk of environmental contamination.

Kujawska et al. [120] conducted phytotoxicity tests to evaluate the effects of biochar on plant germination. The results showed that biochar produced at a lower pyrolysis temperature (400 °C) promoted plant growth when applied at rates of 5 and 10 t ha^−1^. In contrast, biochar produced at a higher pyrolysis temperature (800 °C) exhibited significant phytotoxicity.

To reduce the environmental exposure and risks associated with heavy metals, several mitigation strategies have been proposed, including optimization of pyrolysis operating conditions, the addition of kaolin or phosphoric acid to reduce the volatilization of heavy metals, and the implementation of flue gas cleaning systems during pyrolysis [108].

Contrarily, Wang et al. [111] reported that direct pyrolysis markedly reduced heavy metal leaching, thereby lowering the ecological risks associated with sewage sludge.

In another study [121], twelve heavy metals (Cr, Cu, Zn, Mn, Ni, Ba, As, Se, Hg, Pb, Be, and Cd) were detected in a biochar produced from sewage sludge obtained from a WWTP in China. Although pyrolysis increased the total concentrations of heavy metals due to the loss of organic matter, it significantly reduced their mobility and leachability by converting them into more stable chemical forms. More recently, co-pyrolysis and composite fabrication strategies have been developed to overcome the limitations of single-component sludge-derived biochar by integrating complementary functional materials.

Embaye et al. [121] investigated the co-pyrolysis of sewage sludge with mixed municipal solid waste at a pyrolysis temperature of 400 °C. The authors found that at a sewage sludge to municipal waste ratio of 50:50, reduced Se leaching from 1.54% to 0.12%. Ecological risk (Er) assessment further demonstrated significant reductions in the risks associated with Hg and Cd of 86.6 and 41.23, respectively, resulting in a decrease in the cumulative ecological risk (RI) from 339.7 to 175.7. Similarly, Mohamed et al. [122], who performed an LCA for co-pyrolysis of sewage sludge mixed with wood or corncobs, reported that the overall environmental impacts were reduced by up to 48%. In particular, the increase of mixing biomass ratio to 50 and 75% had a favorable effect on global warming and decreased the overall negative environmental impacts.

### 4.4. Practical Applications

Table 6 compares the advantages, limitations, and practical applications for investigated advanced oxidation processes, technologies-based membranes, and hybrid treatment systems used for purification of landfill leachates.

Data from Table 6 showed that the hybrid systems that combine biological treatment, adsorption, advanced oxidation, and membrane separation generally provided the highest overall treatment efficiency.

Among biological treatment technologies, membrane bioreactors (MBRs) have emerged as one of the most effective options for landfill leachate treatment, consistently achieving removal efficiencies exceeding 90% for BOD, COD, and NH_3_-N. In addition to their high treatment performance, MBRs require smaller footprints, produce less excess sludge, and exhibit lower membrane fouling than many conventional biological systems [63]. However, these advantages are associated with considerable capital and operating costs. Initial investment typically ranges from USD 7700 to USD 17,000 per m^3^/day of treatment capacity, while full-scale facilities require approximately 5–25 m^2^ per m^3^/day of wastewater treated. Operating costs average USD 3.5–5.0 per m^3^/day, with energy consumption accounting for 43% of total expenses, followed by chemicals (19%) and membrane replacement (17%) [63]. Consequently, the implementation of MBR technology requires balancing its superior treatment efficiency against its economic and spatial requirements.

Su et al. [90] showed that the addition of wheat dust biochar (WDB) to polyferric sulfate (PFS) during the treatment of leachate membrane concentrate (LMC) significantly enhanced sludge dewaterability. After 15 h of settling, the final sedimentation ratio was 19.8% lower than that obtained with coagulation alone. For a treatment plant with an LMC capacity of 100 m^3^/day, this improvement corresponds to a daily sludge reduction of 19.8 m^3^ and an annual reduction of 7227 m^3^, resulting in estimated cost savings of approximately CNY 2.44 million per year.

Nature-based solutions incorporating biochar have also demonstrated promising practical applications [54,56,57]. Wei et al. [54] proposed the use of yard waste (YW) biochar in constructed wetlands (CWs), where it simultaneously served as a nutrient source for microbial growth and an adsorbent for total dissolved solids (TDS). Application of YW biochar at a loading rate of 10 kg/m^2^ resulted in the retention of approximately 1000 mg of total nitrogen per m^2^ of wetland bed and achieved a TOC adsorption capacity of 2.1 mg/g.

Further improvements have been achieved by integrating constructed wetlands with membrane treatment for water reuse. Lam et al. [57] evaluated four pretreatment strategies for landfill leachate generated in Florida prior to an ultrafiltration–reverse osmosis (UF–RO) system, including activated sludge, conventional hybrid CWs, and adsorbent-enhanced CWs amended with zeolite and biochar. The incorporation of these adsorbents substantially improved leachate quality, reducing turbidity from 86.3 to 1.58 NTU, NH_4_^+^-N from 367 to 46.5 mg/L, COD from 782 to 273 mg/L, and BOD_5_ from 29.5 to 1.7 mg/L, corresponding to a 94.2% BOD_5_ removal efficiency. By mitigating membrane fouling, the integrated adsorbent-enhanced CW–UF–RO system also reduced the net present value cost from USD 831,000 to USD 282,000 per m^3^/day of landfill leachate treated, making it a sustainable alternative to transporting leachate to municipal WWTPs. Moreover, the treated effluent met the quality requirements for on-site reuse in irrigation and industrial applications, demonstrating the practical potential of integrated nature-based and membrane technologies for circular water management.

## 5. Prospects

In landfill leachate treatment, different process configurations are commonly applied depending on influent characteristics.

Adsorption processes, although highly effective as polishing or post-treatment units for residual organic contaminants, are limited by finite adsorption capacity, adsorbent saturation, and the need for regeneration or replacement, which may increase operational costs. Improvements in the adsorption efficiency of sewage sludge-derived biochars for landfill leachate treatment remain an important research direction, particularly under continuous bench-scale conditions, where scale-up analyses can further support process optimization and design [29].

The membrane bioreactor (MBR) technology provides high effluent quality and effective ammonia nitrogen removal due to the coupling of biological degradation with membrane separation; however, its widespread application is constrained by severe membrane fouling, high energy demand for aeration and membrane scouring, and sensitivity of microbial communities to toxic and refractory compounds present in leachate. Consequently, careful optimization of operating conditions, including pH management, is essential to maintain membrane performance and ensure the sustainability of decentralized leachate treatment systems. In comparison, A/O systems are operationally robust and less energy-intensive, but they generally require larger reactor volumes and HRTs, while achieving comparatively lower removal efficiency for refractory organic matter. Reverse osmosis (RO) offers excellent rejection of dissolved salts, organic micropollutants, and color, making it a powerful polishing step; nevertheless, its application is limited by high hydraulic pressure requirements, significant energy consumption, and the generation of a concentrated brine stream that necessitates further treatment or disposal.

Integrated treatment approaches combining biological processes, membrane separation, and adsorption are generally required to achieve stable performance and regulatory compliance. Cong et al. [123] proposed a sludge valorization concept demonstrating that a vertical baffled reactor promoted aerobic sludge concentration and improved sludge quality. These sludge-based adsorbents can subsequently be applied to the treatment of landfill leachate, thereby integrating wastewater treatment, waste minimization, and resource recovery within a circular economy framework.

However, several gaps remain in this field, such as insufficient long-term regeneration studies, concerns about contaminant leaching and the fate of spent adsorbents, and the need for more comprehensive techno-economic and life-cycle assessments (LCA). Addressing these issues is essential before sludge-based adsorbents can be widely adopted in full-scale landfill leachate treatment systems. The desorption and regeneration of sewage SBAs remain challenging because of their low recovery efficiency and the generation of secondary pollution from regeneration solutions [95]. A few studies reported regeneration studies of biosorbents [4,91]. Future research should also focus on the development of magnetic adsorbents, owing to their high reusability, excellent stability, and favorable adsorption capacity [124]. Cui et al. [95] proposed the use of spent SBAs as soil amendments or fertilizers, fuels, electrode materials for supercapacitors, and catalyst supports.

Long-term landfill management strategies also play a critical role in minimizing environmental impacts. Turner et al. [125] investigated the influence of different landfill aftercare strategies on long-term environmental performance, especially when active leachate collection and treatment systems fail or are discontinued. Using a mechanistic leachate-flow model coupled with LCA over a 10,000-year time horizon, the authors showed that the accelerated aftercare strategies may be environmentally preferable to conventional containment-based landfill management. Rather than attempting to keep water out indefinitely, controlled stabilization and contaminant removal can reduce long-term environmental risks and decrease the reliance on centuries of active management. Additionally, the possibility of future failures of leachate control systems should be explicitly considered in landfill sustainability assessments.

Similarly, effective post-closure management depends not only on technical solutions but also on clearly defined institutional responsibilities among municipalities, landfill operators, and regulatory authorities. Petrovic et al. [126] evaluated the management of three closed landfill sites in Norway and found that these sites continue to generate leachate and CH_4_ for decades after their closure. Moreover, even several years after landfill closure, the leachate continues to accumulate and decompose [127]. Alao et al. [128] examined the extent of leachate plume contamination in Nigeria using subsurface resistivity and hydrochemical analyses. The authors found that the soil overlying the groundwater was heavily contaminated due to the infiltration of leachate carrying elevated concentrations of pollutants. Thus, the leachate contained total dissolved solids (TDS) ranging from 400 to 1612 mg/L, biological oxygen demand (BOD_5_) from 371 to 611 mg/L, chemical oxygen demand (COD) from 697 to 1117 mg/L, and heavy metal concentrations between 0.012 and 1.787 mg/L [128].

In addition, reducing the global warming potential (GWP) of treatment systems requires the use of renewable natural resources and greener synthesis routes for adsorbents. The LCA results indicated the relative environmental impacts associated with natural AC adsorbents activated with HCl and NaOH. The analysis revealed that HCl emissions were the major contributor to GWP, accounting for 25%. Other significant environmental impact categories included terrestrial acidification, contributing 38%; freshwater and marine eutrophication, contributing 34%; terrestrial, freshwater, and marine ecotoxicity, contributing 49%; human carcinogenic and non-carcinogenic toxicity, contributing 54%; and mineral resource scarcity, contributing 59%. Furthermore, the regeneration and reuse of the adsorbent significantly improved its environmental sustainability, reducing the global warming impact by 2963 kg CO_2_ eq compared with the production of newly synthesized adsorbents [129].

Besides the recovery of sewage sludge as adsorbents for landfill leachate treatment, a proactive strategy involves enhancing CH_4_ recovery while mitigating GHG emissions. For instance, early characterization of municipal solid waste to identify food waste fractions can enable the design of appropriate landfill gas collection (LGC) systems prior to landfill operation, improving overall environmental performance [22].

Nevertheless, further studies are still required to evaluate long-term stability, regeneration efficiency, and large-scale implementation of SBAs under real landfill operating conditions. Future studies should also prioritize the development and application of artificial intelligence (AI) and machine learning (ML) approaches to predict treatment performance and optimize landfill leachate treatment processes, with the aim of facilitating their scale-up for practical applications [130].

## 6. Conclusions

This review showed that the sludge-based adsorbents (SBAs) produced through the pyrolysis and chemical activation of sewage sludge and municipal solid waste sludge represent a promising and sustainable strategy for removing refractory pollutants from landfill leachate.

The physicochemical properties and adsorption performance of these SBAs can be improved by optimization of the pyrolysis parameters, chemical modification, loading of metal and NPs, co-pyrolysis, as well as the fabrication of hybrid composites. The advances in SBAs have progressed from simple carbonization of sewage sludge to engineered multifunctional materials with tailored porosity, improved surface chemistry, catalytic activity, and enhanced microbial compatibility.

Among physicochemical, biological, and a combination of biological and physicochemical treatments, those including sequencing batch reactors (SBRs) coupled with aquatic plant-assisted remediation, membrane bioreactors (MBRs), as well as magnetic biochar nanocomposites were found to have the greatest potential for the simultaneous removal of different organic and inorganic pollutants from landfill leachate.

This review provides opportunities for SBAs to support sustainable landfill leachate management while promoting sludge valorization and resource recovery within a circular economy framework.

Future research should focus on optimizing SBA synthesis, evaluating long-term stability and regeneration, assessing environmental and economic performance through life cycle assessment (LCA) and techno-economic analysis (TEA), and validating these technologies under full-scale operating conditions to facilitate their practical implementation.

## Figures and Tables

**Table 1:** Comparison of existing review articles with the present study, highlighting research scope and knowledge gaps.

Review	Year	Focus	Main Finding	Gap Addressed by This Review
Hu et al. [44]	2022	Sludge-derived biochar	Preparation and modification methods for contaminant removal in water	Modification strategies, adsorption/catalytic removal mechanisms, and perspectives; Limited discussion of landfill leachate
Mushtaq et al. [45]	2024	Sludge-based activated carbon	Preparation and application of sludge-based activated carbon for the treatment of landfill leachate	Removing heavy metals, textile dyes, phosphorus and phosphates; Parameters that influence the adsorption mechanism; Limited to activated carbon-based adsorbents
Wang et al. [46]	2018	Landfill leachate treatment technologies	Biological treatment of landfill leachate	Demonstrated the effective removal of organic matter, ammoniacal nitrogen, and total nitrogen (TN) with activated sludge; Did not discuss the comparative evaluation of sludge-based adsorbents
Râpa et al. [47]	2024	Circular economy approaches to waste valorization	Valorization of fruit and vegetable waste; Valorization of root vegetable waste; Circular wastewater valorization; Closed-loop water reuse systems; Conversion of olive pomace by thermochemical processes; Valorization of apple pomace	Value-added products include bioactive compounds, biopolymers, bioenergy, and materials for environmental applications; Green extraction technologies; Techno-economic considerations
Matei et al. [48]	2026			Integrated biorefinery; Circular bioeconomy strategy
Bosco et al. [49]	2025			Recovery of water, materials, energy, and nutrients
Sharma et al. [50]	2024			Advanced treatment technologies; Economic and environmental sustainability; Regulatory and implementation challenges
Berenguer et al. [51]	2023			Producing bio-oil, gas, and hydrochar by pyrolysis; Synthesis of hydrochar for fuel and agricultural applications through hydrothermal carbonization (HTC)
Ozkan et al. [52]	2023			Functional food ingredients; Antioxidant-rich nutraceuticals; Biodegradable packaging materials
Ukoha-Onuoha and Horsfall [53]	2026			Wastewater treatment technologies for agricultural reuse; Nutrient recovery
Present review		Sludge-based adsorbents	Synthesis of sludge-based adsorbents; Performance; Circularity of landfill leachate	Comprehensive comparison of sludge-derived adsorbents for the treatment of landfill leachate

**Table 2:** Comparison of the discharge limits for the various landfill leachates treated.

Parameter	USA, Maximum Daily [71]	USA, Maximum Monthly Avg. [71]	China [78]	Malaysia [9,83]	Morocco [61]
pH	6.0–9.0	6.0–9.0	6.0–9.0	6.0–9.0	5.5–8.5
Color (Pt-Co)	-	-	-	100	-
COD (mg O_2_/L	-	-	100	400	120
BOD_5_ (mg O_2_/L)	140	37	30	20	40
TKN (mg N/L)	-	-	40	-	40
NH_3_-N (mg/L)	10	4.9	25	5	-
TP (mg P/L)	-	-	3	-	2
TSS (mg/L)	88	27	30	50	30
EC (µS/cm)	-	-	-	-	2700
Fe (mg/L)	-	-	-	5	-
Cu (mg/L)	-	-	40	0.2	-
Mn (mg/L)	-	-	-	0.2	-
Ni (mg/L)	-	-	0.5	0.2	-
Pb (mg/L)	-	-	0.25	0.1	-
Zn (mg/L)	0.2	0.11	100	2	-
Cd (mg/L)	-	-	0.15	0.01	-
Se (mg/L)	-	-	-	0.02	-
Cr(VI) (mg/L)	1.1	0.46	0.05	0.05	-

**Table 3:** Performance of various methods used for the treatment of landfill leachate.

Sample	Characterization	Method	Optimal Parameters	Performance	Reference
Leachate sample (Malaysia)	pH = 8.19 ± 0.11; NH_3_-N = 1765.34 ± 190.54 mg/L; COD = 2739.06 ± 225.68 mg/L; BOD_5_ = 249.45 ± 61.51 mg/L; Fe = 7.19 ± 0.93 mg/L; Color = 4539.56 ± 260.00 Pt-Co	Adsorption (Peat mixed with AC)	100 mL of leachate; Peat:AC ratio = 1:1 for color and 2.5:1.5 for Fe; pH = 7; 2 h and a speed of 200 rpm	Fe = 79.6%; Color = 74.4%	[39]
Pre-treated landfill leachate collected prior to the tertiary treatment (Malaysia)	COD = 370 mg/L; NH_3_-N = 38.5 mg/L; TKN = 385.5 mg/L; Cr = 0.608 mg/L; Cu = 0.18 mg/L; Fe = 8.659 mg/L; Ni = 0.576 mg/L	Adsorption (Biochar from wood waste (WCBC))	WCBC-40; Adsorbent dose = 60 g/L; 150 rpm for 24 h in a jar test	COD = 73%; NH_3_-N = 70%; TKN = 97%; Cr = 99.8%; Cu = 94.4%; Fe = 98.5%; Ni = 99.4%	[83]
Real landfill leachate, synthetic landfill leachate (USA)	COD = 6000 mg/L	Adsorption (Biochar from organic fraction of municipal solid waste (OFMSW))	Biochar doses = 25 to 728 mg/L and 120 to 6500 mg/L	Nitrobenzene and 2,4-D = 50% and 25%, respectively	[89]
Leachate membrane concentrate collected from the waste incineration plants	pH = 7.9 ± 0.1; TOC = 2141 ± 327 mg/L; NH_4_^+^-N = 104.38 ± 21.02 mg/L; Ca^2+^ = 1383 ± 401 mg/L; Chroma = 6.06 ± 0.25 cm^−1^	Coagulation (Polyaluminum ferric silicate (PSAF)) and adsorption (wheat dust biochar (WDB))	pH = 5.9; Coagulant dosage = 4.8 g/L; Adsorbent dosage = 1 g/L; Temperature = 19.9 °C; Stirring speed = 400 rpm; Stirring time = 15 min (RSM—optimized conditions)	TOC = 75.20% (in the presence of coagulant); TOC = 81.36%; NH_4_^+^-N = 82.85%; Ca^2+^ = 90.23%; Chroma = 95.87% (in the presence of biochar)	[90]
Stabilized Leachate (Malaysia)	COD = 1712 ± 282.51 mg/L; BOD_5_/COD = 0.07 ± 0.02; NH_3_-N = 405.37 ± 1.61 mg/L; pH = 7.96 ± 0.33	Adsorption (Green mussel (*Perna viridis*))	Speed = 200 rpm; pH = 7; Adsorbent dosage = 2 g	COD = 65%; NH_3_-N = 45%	[7]
Phosphogypsum (PG) leachate (Turkey)	Cd = 1.1 mg/L; Cr = 17.3 mg/L; Co = 11.3 mg/L; Cu = 0.8 mg/L; Hg = 0.7 mg/L; Mn = 20.5 mg/L; Mo = 2.9 mg/L; Ni = 6.7 mg/L; Pb = 3.6 mg/L; Zn = 2.1 mg/L	Adsorption (nGO, MWCNT-COOH, and MWCNT-COOH-Fe_3_O_4_ carbonaceous materials)	Solid-to-liquid ratios of 1:1, 2:1 and 3:1; pH = 5.0–5.8; 300 rpm for 120 min; Temperature = 35 °C	Cd = 80%; Co = 76%; Mn = 69%; Zn = 76%; At 1/3 PG/MWCNT-COOH-Fe_3_O_4_ ratio:Cr = 82%; Cu = 83%; Ni = 78%; Pb = 88%; nGO and MWCNT-COOH-Fe_3_O_4_ at 35 °C: Fe = 76%	[6]
Synthetic and real municipal landfill leachate (Canada)	COD = 920–1050 mg O_2_/L; Color = 1590–2222 Pt-Co units	Adsorption (Oat hulls activated carbon and pyrolyzed for 1 h (N_2_ atmosphere) at 500 °C)	Adsorbent dose = 20 g/L; pH = 4; Temperature = 20 °C; Contact time = 3 h	Synthetic leachate: Q_e_ = 27 mg/g (at 100% impregnation); Real leachate: Q_e_ = 40 mg/g; Color = 100%; COD = 90%	[8]
Sample collected from a municipal sanitary landfill leachate (Turkey)	COD = 16,000 ± 1500 mg/L, DOC = 7058 ± 400 mg/L, NH_4_^+^-N = 2120 ± 200 mg/L, NO_3_^−^ = 670 ± 40 mg/L	Adsorption (Nano zero-valent iron (nZVI))	Adsorbent dose = 50 mg/L; pH = 8; contact time = 120 min.	COD = 75%; DOC = 60%; NH_4_^+^-N = 57%; NO_3_^−^ = 33%	[59]
Landfill leachate (Iran)	COD = 12,580 mg/L	Adsorption (Magnetic GO–Fe_3_O_4_/WO_3_ nanocomposite)	Adsorbent dosage = 25 mg/L; pH = 4; contact time = 90 min; T < 298 K	Q_max_ = 3561.83 mg COD/g; COD removal ≈ 71%; 57% adsorption capacity after five regeneration cycles (0.1 M HCl)	[91]
Pilot landfill leachate (China)	pH = 3.8–4.2; COD = 90,000–100,000 mg/L; VFAs = 1800–2200 mg/L; UV_254_ = 45–55 cm^−1^	Adsorption (S,N-modified biochar (waste bamboo strips, urea, and thiourea))	Dose = 1 g/L; Temperature of 35 °C; Hydraulic re-tention time (HRT) = 24 h; Operation time = 30 days	COD = 91%; VFAs = 64%; UV_254_ = 36.5%	[60]
Municipal sanitary landfill leachate mixed with wastewater and activated sludge resulted (WWTP in Malaysia)	Fe = 6.03 mg/L; Mn = 1.98 mg/L; Ni = 4.94 mg/L; Cd = 2.71 mg/L, Color = 1690 Pt-Co; COD = 1301 mg/L; NH_3_-N = 532 mg/L	Sequencing batch reactor (SBR) coupled with powdered ZELIAC (PZ) adsorbent	Aeration rate/ Contact time/Leachate to wastewater ratio For total Fe = 1.71 L/min/12.87 h/25.73% (according to RSM); For Mn: 3.92 L/min/7.71 h/20.69%; For Ni: 3.22 L/min/9.15 h/20.22%; For Cd: 3.68 L/min/15.31 h/20.03%	Fe = 80.06%; Mn = 73.38%; Ni = 79.10%, Cd = 76.59%	[9]
Mature leachate collected from seven landfill sites (Sri Lanka)	pH ranging from 7.1 to 8.3; Electrical conductivity (EC) values between 5 and 25 mS/cm; BOD_5_/COD ratio < 0.1	Anaerobic MBRs	1 L leachate Sludge retention time (SRT) = 60 days; HRT = 24 h	BOD_5_ = 93%; COD = 64.8%; TN = 59%	[63]
Biologically pretreated landfill leachate (Sri Lanka)		A/O MBR coupled with a tertiary adsorption unit	pH = 2; Adsorbent dosage = 20 g/L; Contact time = 48 h; Temperature = 25 °C; Speed = 200 rpm	BOD_5_ = 258 mg/L; COD = 650 mg/L; PO_4_^3−^ = 86 mg/L; NO_3_^−^ = 206 mg/L; NO_2_^−^ = 0 mg/L; SO_4_^2−^ = 242 mg/L; Zn = 1.056 mg/L; Pb = 0.012 mg/L; Color = 96 ± 3.8%; Q_e_ = 123.8 Pt–Co/g; Three cycles of regeneration	[4]
Leachate from controlled landfill (Morocco)	COD = 10,805 mg/L; BOD_5_ = 1530 mg/L; TKN = 5253 mg/L; NH_4_^+^ ≈ 4827 mg/L; TP = 58.6 mg/L; TSS = 55.7 mg/L; pH = 8.5; EC = 29.3 mS/cm	Biological pre-treatment combined with reverse osmosis (RO)	Temperature = 19–20 °C; Dissolved oxygen = ~2–3 mg/L; pH = 8.5	COD = 99.31%; BOD_5_ = 97.4%; NH_4_^+^ = >99%; TP = 99.27%; LPI < 10	[61]
Landfill leachate (China)	pH = 7.2 ± 0.14; COD = 12,320 ± 537.40 mg/L; NH_4_^+^-N = 1583.16 ± 101.03 mg/L; UV_254_ = 16.99 ± 0.73	Struvite precipitation process, O_3_ catalytic oxidation, and post-MBR	Peroxymonosulfate (PMS) oxidation = 1500 mg/L with 1360 mg/L Fe^2+^ (ratio (PMS):(Fe^2+^) = 1:1) for 1 h; O_3_ direct oxidation = 20 g/h aeration for 1 h	COD = 91.2%; NH_4_^+^-N = 99.4%; UV_254_ = 12.2	[86]
Concentrated leachate resulted from reverse osmosis (RO) and leachate treatment plant and activated sludge (WWTP from China)	Color = 2113 Pt-Co; BOD_5_ = 285 mg/L; COD = 3018 mg/L; BOD_5_/COD = 0.09; NH_3_-N = 49.00 mg/L; Ni = 29.67 mg/L	Electro-ozonation combined with a BAZLSC composite adsorbent augmented sequencing batch reactor (SBR) process (PB-SBR)	Reaction time = 96.9 min; O_3_ dosage = 120.0 mg/L; pH = 7.3; Current intensity = 4 A; Voltage = 9 V	COD = 88.2%; Ni = 73.4%; Color = 96.1%	[23]
Landfill leachate (China)	COD = 720–800 mg/L; TOC = 140–160 mg/L; NH_3_-N = 240–270 mg/L; TP = 6.1–7.0 mg/L; TN = 320–370 mg/L	Magnetic coconut shell biochar (MCSB); Activated sludge collected from WWTP; Anaerobic/Aerobic (A/O) biological system	Adsorbent dose of 5 g/L; HRT of 3 days	TOC = 80.01% and 76.36% in A/O systems; TN = 65.27% and 50.26% removal rates in A/O systems; Tetracycline antibiotics (TCs) = 81–88%	[14]

**Table 4:** Treatment of landfill leachate using sewage sludge-based adsorbents.

Sample	Characterization	Method	Optimal Parameters	Performance	Reference
Pre-settled landfill leachate (Iran)	COD = 3467 ± 254; BOD_5_ = 1507 ± 137; TKN = 127.5 ± 12.1 mg/L	Hybrid coagulation–MWAS adsorbent	pH = 8; adsorbent dose = 12 g L^−1^; contact time = 120 min	BOD_5_ = 69.13%; COD = 78.68%; TKN = 69.45%; TP = 41.53%; TSS = 61.01%	[96]
Integrated CWs with conventional activated sludge treatment (Poland)	pH = 8.3; EC = 6020 ± 120–7150 ± 715 µS/cm; TP = 2.21 ± 0.04–1.85 ± 0.37 mg/L; COD = 1100 ± 165–620 ± 124 mg O_2_/L	SBR coupled with aquatic plant-assisted remediation, adsorption, and ion exchange	138 days; 24 h cycle (0.5 h of filling, 3 h of mixing, 19 h of aeration, 1 h of sedimentation activated sludge, 0.5 h of automatic decantation of treated leachate); Temperature in the range of 20–22 °C; Plant density of 150 plants/m^2^	EC = 4.15–23.08 µS/cm; TP = 100%; COD = 10.9–44.52 mg O_2_/L	[56]
Old sanitary landfill leachate (Brazil)	COD = 1315 mg/L; DOC = 487 mg/L; Apparent color = 3000 CU	Adsorption using biochars produced from aerobic and anaerobic sludge	Adsorbent dose of 20 g/L	COD = 21.5 ± 1.7% and 32.3 ± 1.1% for aerobic and anaerobic biochar, respectively; DOC = 28.1 ± 1.0% and color removal = 24.1 ± 1.2% for aerobic biochar; DOC = 36.1 ± 0.3% and color removal = 41.3 ± 1.4% for anaerobic biochar	[29]
Old landfill Leachate (Chile)	pH = 8.8 ± 0.2; COD = 20,603.0 ± 6909.4 mg/L; NH_4_^+^-N = 1889.6 ± 32.2 mg/L; NO_3_−N = 187.2 ± 23.8 mg/L; Mn = 104.6 ± 6.5 mg/L; Fe = 252.3 ± 44.2 mg/L; Zn = 2.9 ± 2.2 mg/L	Aerobic granular sludge (AGS)	Reactor volume = 2.0 L and T = 30 °C; 50-day start-up period; A 148-day stabilization phase	COD = 85%; TN = 27%; Cu = 93.6%; Fe = 50.3%; Mn = 12.4%; Zn = 77.3%; Pb = 92.9%	[97]
Synthetic landfill leachate (China)	pH = 7.2 ± 0.4; COD = 1800 mg/L; NH_4_^+^-N = 280 mg/L; TN = 300 mg/L; PO_4_^3−^-P = 20 mg/L; BPA = 20 mg/L; Humic acid (HA) = 60 mg/L	Mg–Fe LDH-Biochar composite synthesized through co-precipitation method; Sequence batch reactors (SBR)	Dose of inoculated sludge = 8 g/L; Biochar dose = 0.5 g/L; DO concentration = 6–8 mg/L; STR = 30 days	PO_4_^3−^-P = 94.55% at 1 g/L; TN = 78.65%; Humic acid = 94.34%; Bisphenol A (BPA) = 96.8%	[13]
Mature leachate (China)	pH of 8.4 ± 0.4; NH_4_^+^-N = 1078.3 ± 212.1 mg/L; NO_2_^–^-N = 0.4 ± 0.1 mg/L; NO_3_^–^-N = 156.8 ± 30.6 mg/L; COD = 3578.5 ± 768.1 mg/L; TN = 1376.5 ± 177.0 mg/L; TP = 8.8 ± 1.9 mg/L; Alkalinity = 11,783.6 ± 575.6 mg/L	Anammox technology; Biochar/nZVI modified polyurethane biocarrier (BZPU) (1:1 mass ratio of biochar to nZVI)	207 days; Four stages; Temperature in the range of 27–30 °C	Nitrogen removal rate (NRR) = 0.76 kg N/(m^3^ × d); Total nitrogen removal rate (TNRE) = 92.0%	[98]
Mature landfill leachate (China)	pH = 8.26; NH_4_^+^-N = 1951 ± 2.01 mg/L; COD = 698 ± 73.54 mg/L; NO_3_^−^-N = 22.99 ± 0.16 mg/L	MBR-ozone-catalyzed internal circulation	25 L of leachate, Dosage of biochar of 5 g/L; Ozone aeration rate of 10 g _O3_/h; HRT = 20 h; Aeration intensity of 160 L/h; Dissolved oxygen (DO) = 7 mg/L	NH_4_^+^-N = 99.6%; COD = 96.9%	[99]
Mature land-fill leachate (China)	pH = 8.9; NH_4_^+^-N = 1087.02 ± 9.56 mg/L; COD = 2290 ± 14.14 mg/L; NO_3_^−^-N = 33.07 ± 0.16 mg/L; UV_254_ = 14.14 ± 0.17	Mn-biochar and catalytic ozonation degradation	25 L of raw leachate; Adsorbent dose of 5 g/L; Aeration intensity of 160 L/h; Ozone aeration rate of 10 g O_3_/h; Operating 14 days; Catalytic ozonation/aeration time = 5 min on/5 min off; Acetic acid dosage of 82.5 mL	NH_4_^+^-N = 99.2%; COD = 96.2%; NO_3_^−^-N = 29.6%; UV_254_ = 98.8%	[100]
Synthetic acetogenic landfill leachate	COD = 6520–8700 mg/L; NH_3_-N = 510–544 mg/L; Cd(II) = 32 ± 2 mg/L	FeO NPs-coated sewage sludge (SS) + CTAB-modified bentonite (50:50)	5 g/50 mL; 120 min; 200 rpm	>90% removal (batch); ≥85% removal of Cd(II), COD and NH_3_-N (column conditions); Q_e_ for Cd, COD, and NH_3_-N = 0.556, 102, and 10.42 mg/g, respectively	[55]
Stabilized and young municipal waste leachates (Iran)	pH = 7.8–7.1 COD = 13,800/70,375 mg/L; BOD_5_/COD = 015/0.42; Fe = 42.2/9.3 mg/L; Zn = 2.42/0.16 mg/L	Fe NPs; FeCl_3_ sludge; Fresh FeCl_3_	Temperature of 35 ± 2 °C; Fe-NPs = 17.06 mg/L; FeCl_3_ = 0.28 mg/L; Fresh FeCl_3_ = 1.46 mg/L (Box–Behnken design)	COD = 73.64%; Biogas production = 2990 mL	[101]

**Table 5:** Limit values for concentrations of heavy metals for use in agriculture and soil in European countries [69].

Heavy Metal	In Agriculture (mg/kg of Dry Matter)	In Soil (mg/kg of Dry Matter)
Cd	20–40	1–3
Cu	1000–1750	50–140
Ni	300–400	30–75
Pb	750–1200	50–300
Zn	2500–4000	150–300
Hg	16–25	1–1.5

**Table 6:** Advantages, limitations, and practical applications for treating landfill leachates.

Sludge-Based Adsorbent	Advantage	Limitation	Practical Application	Reference
Membrane bioreactors (MBRs)	Stable removal of COD and ammonia under tropical conditions; Compact footprint; Different configurations provide operational flexibility	Membrane fouling; High energy demand; Membrane replacement costs	Municipal landfill leachate treatment plants in tropical climates requiring reliable biological treatment	[63]
Membrane bioreactor (MBR) coupled with catalytic ozonation	High COD removal; Enhanced ozone utilization; Effective for mature leachate with low C/N ratio; Improved degradation of refractory organic matter	Catalyst preparation increases cost; Possible metal leaching; Ozone generation is energy-intensive; Catalyst regeneration required	Advanced polishing of biologically treated mature landfill leachate, especially for low C/N wastewater prior to discharge or reuse	[99]
Magnetic biochar combined with catalytic ozonation	Converts excess sludge into value-added catalyst; High catalytic activity; Efficient removal of recalcitrant organics; Promotes circular economy; Easy to recover	Catalyst stability and Mn leaching require long-term evaluation; Ozone operating costs remain significant; Scale-up needs validation; Limited for heavy metals removal	Sustainable advanced oxidation for landfill leachate treatment while simultaneously recycling wastewater sludge	[100]
Biological pre-treatment combined with reverse osmosis	Excellent pollutant removal; Treated effluent complies with discharge standards; Significantly lowers landfill pollution index (LPI)	RO produces concentrated brine requiring disposal; Membrane fouling; Relatively high capital and operating costs	Integrated treatment plants where stringent discharge or water reuse standards are required	[61]
Adsorbent-enhanced constructed wetlands (CW)-UF/RO	Integrates natural and membrane processes; High removal efficiency; Enables water reuse; Reduced environmental impact; Reduced net present value cost with 66%	Membrane fouling; Wetlands require relatively large land area and longer hydraulic retention time	Sustainable landfill leachate reclamation for non-potable reuse (for example, irrigation, industrial uses)	[57]
Mg–Fe layered double hydroxide (LDH)-biochar coupled with a sequencing batch reactor (SBR)	Efficient for nitrogen, bisphenol A (BPA), PO_4_^3−^-P, and humic acid removal; Reduces aeration demand compared with conventional nitrification-denitrification	Biochar properties strongly influence performance; Long-term stability and replacement frequency require further study; Optimization needed for full-scale systems	Energy-efficient biological nitrogen removal in landfill leachate treatment, particularly for mature leachates with high ammonium concentrations	[13]
Sequential batch reactors (SBRs) coupled with aquatic plant-assisted remediation, adsorption, and ion exchange	Improves adsorption and biological activity; Supports plant growth; Evaluates long-term substrate changes; Environmentally friendly	Adsorption capacity decreases with aging; Substrate replacement/regeneration may be needed; Performance depends on operating conditions	Nature-based leachate treatment systems and constructed wetlands enhanced with biochar/zeolite media	[56]

## Data Availability

No new data were created or analyzed in this study. Data sharing is not applicable to this article.
